# Gut microbiome responds to alteration in female sex hormone status and exacerbates metabolic dysfunction

**DOI:** 10.1080/19490976.2023.2295429

**Published:** 2023-12-28

**Authors:** Tzu-Wen L. Cross, Abigayle M. R. Simpson, Ching-Yen Lin, Natasha M. Hottmann, Aadra P. Bhatt, Samuel J. Pellock, Erik R. Nelson, Brett R. Loman, Matthew A. Wallig, Eugenio I. Vivas, Jan Suchodolski, Matthew R. Redinbo, Federico E. Rey, Kelly S. Swanson

**Affiliations:** a Department of Nutrition Science, Purdue University, West Lafayette, IN, USA; b Division of Nutritional Sciences, University of Illinois Urbana-Champaign, Urbana, IL, USA; c Department of Animal Sciences, University of Illinois Urbana-Champaign, Urbana, IL, USA; d Department of Bacteriology, University of Wisconsin-Madison, Madison, WI, USA; e Cardiovascular Research Center, University of Wisconsin-Madison, Madison, WI, USA; f Department of Chemistry, University of North Carolina, Chapel Hill, NC, USA; g Departments of Biochemistry & Biophysics, Microbiology & Immunology, and The Integrated Program for Biological and Genome Science, University of North Carolina at Chapel Hill, Chapel Hill, NC, USA; h Department of Molecular and Integrative Physiology, University of Illinois Urbana-Champaign, Urbana, IL, USA; i Cancer Center at Illinois, University of Illinois Urbana-Champaign, Urbana, IL, USA; j Carl R. Woese Institute for Genomic Biology-Anticancer Discovery from Pets to People, University of Illinois Urbana-Champaign, Urbana, IL, USA; k Beckman Institute for Advanced Science and Technology, University of Illinois Urbana-Champaign, Urbana, IL, USA; l Veterinary Medicine and Biomedical Sciences, Texas A&M University, College Station, TX, USA

**Keywords:** Gut microbiota, estrogen, ovarian deficiency, menopause, metabolic health

## Abstract

Women are at significantly greater risk of metabolic dysfunction after menopause, which subsequently leads to numerous chronic illnesses. The gut microbiome is associated with obesity and metabolic dysfunction, but its interaction with female sex hormone status and the resulting impact on host metabolism remains unclear. Herein, we characterized inflammatory and metabolic phenotypes as well as the gut microbiome associated with ovariectomy and high-fat diet feeding, compared to gonadal intact and low-fat diet controls. We then performed fecal microbiota transplantation (FMT) using gnotobiotic mice to identify the impact of ovariectomy-associated gut microbiome on inflammatory and metabolic outcomes. We demonstrated that ovariectomy led to greater gastrointestinal permeability and inflammation of the gut and metabolic organs, and that a high-fat diet exacerbated these phenotypes. Ovariectomy also led to alteration of the gut microbiome, including greater fecal β-glucuronidase activity. However, differential changes in the gut microbiome only occurred when fed a low-fat diet, not the high-fat diet. Gnotobiotic mice that received the gut microbiome from ovariectomized mice fed the low-fat diet had greater weight gain and hepatic gene expression related to metabolic dysfunction and inflammation than those that received intact sham control-associated microbiome. These results indicate that the gut microbiome responds to alterations in female sex hormone status and contributes to metabolic dysfunction. Identifying and developing gut microbiome-targeted modulators to regulate sex hormones may be useful therapeutically in remediating menopause-related diseases.

## Introduction

Sex hormones are one of the main determinants that impact disease susceptibility and prognosis. Women are at significantly great risk of metabolic dysfunction after experiencing menopause, which is an age-related loss of sex hormone production characterized by falling concentrations of the ovarian hormones estrogen and progesterone. Post-menopausal metabolic dysfunction can subsequently lead to numerous chronic illnesses, such as cardiovascular diseases and cancers. Supplementation of estrogen through hormone replacement therapy is generally recommended to prevent menopause-related symptoms and is thought to prevent or limit cardiometabolic and physiological changes occur with menopause. However, conflicting evidence has also shown that hormone replacement therapy is associated with greater risks of cardiac events, coronary heart disease, venous thromboembolic events, dementia, and breast cancers, depending on the types and combinations of hormones, length of time utilized, and medical history.^1–4^ The inconsistency and complexity suggests that the etiology of loss of sex hormone-induced metabolic dysfunction is likely multifaceted.^5^ To date, exact mechanisms by which this age-related loss of sex hormone production leads to metabolic dysfunction remains unclear, which hinders the development of novel therapeutics for women.

The gut microbiota is involved in sex steroid metabolism that can influence sex hormone homeostasis of the host and alters disease development and prognosis. Sex differences in the composition of the gut microbiota are only apparent in adult mice after the onset of puberty, and transplantation of the gut microbiota from male to female mice caused a systemic increase in testosterone and changed the susceptibility to autoimmune diseases.^6^ In humans, greater circulating estradiol concentration has been linked with greater gut microbial diversity, greater relative abundance of *Faecalibacterium*, and lower abundance of *Slackia*, *Butyricimonas*, and Prevotellaceae.^7–9^ Sex steroids are synthesized *de novo* by mammalian cells, yet bacteria are major consumers of steroids in the biosphere^10^ and may modulate sex hormone homeostasis of the host. This host-microbial interaction has been demonstrated through several mechanisms: (1) microbes possessing the enzyme β-glucuronidase can deconjugate steroidal metabolites and allow them to be reabsorbed by the host through enterohepatic circulation;^11,12^ (2) gut bacterial transformation of sterol impact estrogen metabolism;^13,14^ or (3) microbes can catabolize steroids and lead to systemic changes in sex hormone status.^15^ A common colonic bacterium *Bacteroides fragilis* can anaerobically convert estrone into estradiol as well as 16α-hydroxyestrone, an intermediate metabolite in estriol biosynthesis.^13^ Fecal communities can perform multiple bioconversion steps within estrogen metabolism.^13^ Interestingly, gene clusters discovered in *Denitratisoma* sp. strain DHT3 are capable of reversing the conversion of androgens to estrogens, which was thought to be an irreversible step once made in mammalian cells.^14^ Regarding steroid catabolism, the bacterium *Klebsiella aerogenes* isolated from premenopausal women was recently identified to possess 3β-hydroxysteroid dehydrogenase that is able to degrade estradiol and cause a systemic decline of estrogen and induce depression-like behaviors when colonized in mice.^15^ This evidence supports the critical role of the gut microbiome in maintaining sex hormone homeostasis.

Ovariectomy, a commonly used rodent model of human menopause to investigate metabolic dysregulation, has been associated with changes in the composition of the gut microbiome in some studies,^16,17^ while others did not observed differences.^18,19^ Community-level differences of the gut microbiota associated with ovariectomy have been reported, along with specific bacteria taxa changes (e.g., *Turicibacter, Anaeroplasma*, Clostridiaceae, Lachnospiraceae, *Turicibacter, S24–7, and Akkermansia*)^16,20,21^ However, whether these ovariectomy-induced gut microbial perturbations contribute to obesity and metabolic dysfunction remain unclear. A recent study showed that sharing of the gut microbiome through cohousing ovariectomized mice with intact mice did not improve metabolic phenotype of ovariectomized mice.^20^ We speculate that the impact of sex hormone deficiency due to ovariectomy outweighs and masks the gut microbiota-derived impacts on metabolic outcomes. Therefore, herein, we utilized a gonadal-intact gnotobiotic mouse model to determine if the gut microbiome responds to alterations of the ovarian production of sex hormones and exacerbates metabolic dysfunction. We first characterized metabolic phenotypes, inflammation, and longitudinal gut microbiota profiles of ovariectomized mice fed a semi-purified high-fat diet (HFD) along with a well-controlled low-fat diet (LFD) and harvested fecal samples from these “donor mice” for the fecal microbiota transplantation. We then transplanted these fecal samples into germ-free mice (i.e., “recipient mice”) to study the impact of the ovariectomy-associated microbiota on weight gain and metabolic- and inflammation-related outcomes in various tissues. Importantly, male germ-free mice were utilized as recipients to dissociate estrogen-driven impacts.

## Results

### Ovariectomy causes metabolic dysfunction, which is exacerbated by HFD

**Figure 1.**
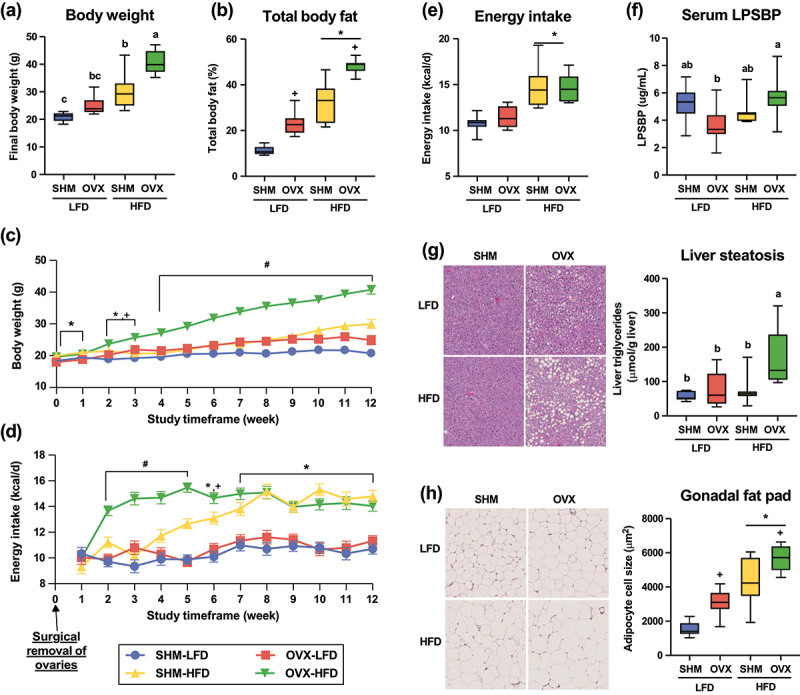
Animal characteristics of conventionally raised C57BL/6J mice that underwent either ovariectomy (OVX) or sham (SHM) surgery and fed either a low-fat (LFD) or a high-fat diet (HFD). Final body weight (a) and energy intake (b) at the end of the 12-week study period as well as body weight (c) and energy intake (d) throughout the study time frame are plotted. Total body fat assessed using EchoMRI (e), serum lipopolysaccharide binding protein (LPSBP) concentration (f), liver steatosis (g), and adipocyte cell size (H) were assessed at the end of the study. *denotes main diet effect; +denotes main surgery effect; interactions are denoted either by superscript letters in box plots or by # sign in line graphs, *p* < .05.

Ten-week-old conventionally raised female C57BL/6 mice were purchased and randomly allotted to consume either a HFD or a LFD for two weeks before undergoing either ovariectomy (OVX) or sham (SHM) surgery as controls. The HFD used in this study was a semi-purified diet that was formulated to provide 60% of the calories using dietary fat sources including lard and corn oil. The LFD was formulated using the same semi-purified ingredients to properly match the HFD in every way possible, except that the proportion of calories coming from dietary fat was 10% and a greater proportion of carbohydrate was necessary to balance caloric needs of the mice. Body weight and food intake were assessed weekly for twelve weeks after the surgery to assess diet by surgery impact on metabolic outcomes. At the end of the twelve-week intervention, mice were euthanized. Detailed dietary composition for both diets is shown in the Supplemental Table S1. Energy intake was calculated based on metabolizable energy of the diet and the amount of food intake recorded. Loss of ovarian hormone production through surgical removal of ovaries led to elevated body weight and fat mass gain, but the magnitude of those fed the HFD was greater than those fed the LFD (Figure 1a–c). Immediately after surgery, OVX-HFD mice began consuming greater (*p* < 0.05) calories than SHM-HFD mice (Figure 1d). However, over time, this difference went away as the caloric intake of SHM-HFD group increased to that similar of the OVX-HFD group. By the end of the study, a main diet effect was observed with HFD-fed mice consuming more calories than LFD-fed animals, while the ovarian hormone status did not affect energy intake (Figure 1d,e).

At euthanasia, liver and adipose tissues were collected to assess severity of steatosis and inflammation using three different methods: (1) a biochemical assay to determine liver triglyceride concentrations; (2) blinded evaluation by a pathologist; (3) calculated adipocyte volume using the cross-sectional area obtained from perimeter tracing. We observed significantly (*p* < .05) higher concentrations of triglyceride in liver of OVX-HFD mice than all other treatment groups (Figure 1g). However, blinded histopathological evaluation suggested that regardless of diet, OVX led to greater hepatic steatosis than SHM while HFD did not exacerbate the severity of steatosis (Supplemental Table S2). Greater (*p* < .05) adipose tissue steatitis was observed in mice fed the HFD compared to those fed the LFD (Supplemental Table S2). A similar diet effect was observed in adipocyte sizing, suggesting that HFD feeding promotes adipocyte hypertrophy, which is associated with cell death and adipose tissue macrophage infiltration and inflammation (Figure 1h). Furthermore, a surgery effect was also observed where OVX mice had greater adipocyte sizing in the gonadal adipose depot than SHM mice.

Blood biomarkers associated with metabolic dysfunction were assessed at euthanasia, 12 weeks post-surgery intervention. A significant (*p* < .05) diet effect, but no surgery effect, was observed with HFD-fed mice having greater fasting circulating glucose and lower alkaline phosphatase concentrations than that of LFD, 12 weeks post ovariectomy (data not shown). Fasting circulating triglycerides, non-esterified fatty acids, aspartate transaminase, and alanine transaminase were not different among treatment groups (data not shown).

### High fat diet and ovariectomy both drive adipose and hepatic inflammation

**Figure 2.**
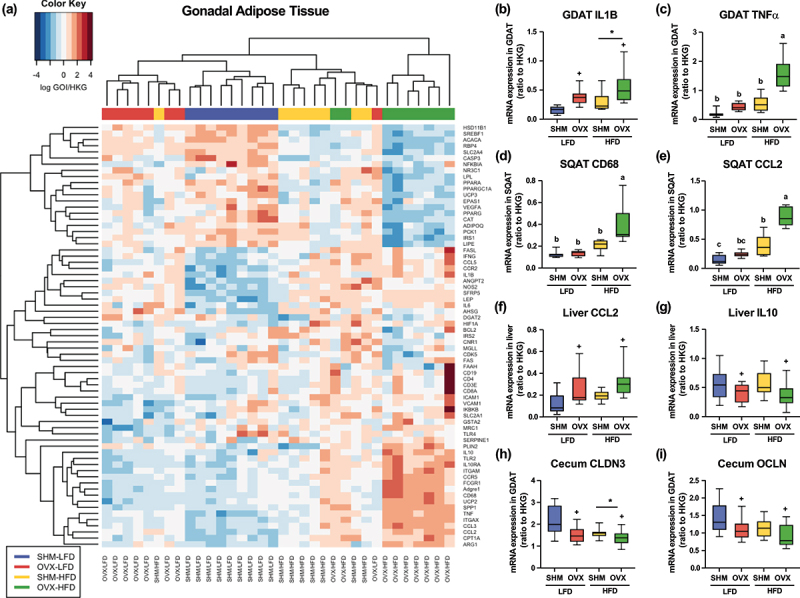
Gene expression profiles of tissues collected from conventionally-raised C57BL/6J mice that underwent either ovariectomy (OVX) or sham (SHM) surgery and fed either a low-fat (LFD) or a high-fat diet (HFD). Hierarchical clustering based on similarity of gonadal adipose tissue (GDAT) gene expression with (a) and relative expression of genes related to inflammation and tight junction proteins in GDAT, subcutaneous adipose tissue (SQAT), liver, and cecum (b-i) . Each row in the heatmap represents a specific gene of interest, and each column represents one sample with color code denoting treatment groups (SHM-LFD: blue; OVX-LFD: red; SHM-HFD: yellow; OVX-HFD: green). *denotes main diet effect; +denotes main surgery effect; interactions are denoted by superscript letters, *p* < .05.

Gene markers associated with inflammation, macrophages, oxidative stress, hypoxia, and energy, lipid, and glucose metabolism were assessed in the adipose tissue depots, liver, and intestines from these mice (see all genes assessed in Supplemental Table S3). Hierarchical clustering of gene expression patterns in gonadal adipose tissue (GDAT) revealed substantial differences among treatment groups (Figure 2a). The HFD mice had greater (*p* < .05) relative expression of IL1B, IL6, and IFNG in GDAT compared to mice fed the LFD (Figure 2a,b). Moreover, OVX mice had an elevated (*p* < .05) relative expression of IL1B and IL6 than SHM mice. OVX-HFD mice had an increased expression of genes associated with inflammatory and macrophage markers, and reduced expression of genes associated with mitochondria biogenesis, glucose uptake, lipolysis, and adipokines [adiponectin (ADIPOQ) and retinol binding protein 4 (RBP4)] in GDAT. The OVX-HFD group had greater (*p* < .05) relative GDAT expression of pro-inflammatory and macrophage markers TNFα, CCL2 (MCP1), CCL3, Adgre1 (F4/80), ITGAM (CD11B), ITGAX (CD11C), CD68, TLR2, compared to all other treatment groups (Figure 2a,c).

Similar to GDAT, substantial modulation of gene expression among treatment groups was observed in subcutaneous adipose tissue (SQAT, Supplemental Figure. S1). However, the response of HFD-fed mice appears to be more variable than those fed LFD. In SQAT, the expression of Adgre1 (F4/80), IL1B, IL6, ITGAM (CD11B), MRC1, NOS2, TLR2, TNF, CCL3, and IKBKB was greater (*p* < .05) in HFD than LFD-fed mice. OVX mice, on the other hand, had lower (*p* < 0.05) relative expression of ARG1 and NFKBIA, and greater (*p* < .05) NOS2, TNF, and CCL3 expression, suggesting that ovariectomy promotes M1 killer phenotype of macrophages and inflammation in SQAT. An interaction was observed for several genes, with the OVX/HFD group having the greatest (*p* < .05) expression of CD68, CCL2, and ITGAX (CD11C) in SQAT among treatment groups (Figure 2d,e).

In contrast, hierarchical clustering of liver gene expression data did not reveal a clear pattern or clustering, suggesting that the metabolic processes impacted in liver by OVX and/or HFD are not as consistent as that of adipose tissues (Supplemental Figure. S2). However, hepatic inflammation associated with loss of ovarian hormones was observed with ovariectomy leading to greater (*p* < .05) expression of the pro-inflammatory marker CCL2 and lower (*p* < .05) expression of an anti-inflammatory maker and its receptor, IL10 and IL10RA compared to SHM mice (Figure 2f,g).

### Ovariectomy disrupts intestinal tight junction protein expression but alterations in permeability may be dependent on both diet and ovarian hormone status

Gene expression of key genes relevant for gut barrier function and lipopolysaccharide binding protein (LPSBP) abundance were used to assess gastrointestinal integrity of the mice (see all genes assessed in Supplemental Table S4). Expression of genes encoding tight junction protein, claudin3 (CLDN3) and occludin (OCLN), was lower (*p* < .05) in the cecum of the OVX group compared to the SHM group (Figure 2h,i). HFD-fed mice had lower (*p* < .05) cecal expression of CLDN3, claudin5 (CLDN5), and claudin8 (CLDN8) than mice fed the LFD (Figure 2h and Supplemental Figure. S3). The expression of tight junction protein-associated genes suggest that gut barrier function may be compromised due to the loss of ovarian hormones or high dietary fat. Gastrointestinal integrity is compromised in obesity and metabolic dysfunction, leading to elevated plasma lipopolysaccharide (LPS), a gut microbiota-derived endotoxin that can promote inflammation and affects host health when it enters the systemic circulation.^22^ In this study, we used LPSBP as a proxy to assess chronic endotoxemia, as measurements for LPS lack sensitivity.^23^ Serum concentration of LPSBP has been associated with HFD, obesity, and chronic diseases.^24–27^ A greater concentration of circulating LPSBP was observed in the OVX-HFD group compared to the OVX-LFD group, but they were not different compared to either of the SHM groups (Figure 1f). Along with the cecal expression of tight junction protein-related genes, our data suggest that the intestinal barrier may be compromised when ovarian hormone production is lost. However, this evidence could be supported by including functional tests in the future, such as a fluorescein isothiocyanate-dextran permeability test.

### Differences of the gut microbiota and fecal β-glucuronidase activity due to ovariectomy were only observed in the LFD-fed mice

**Figure 3.**
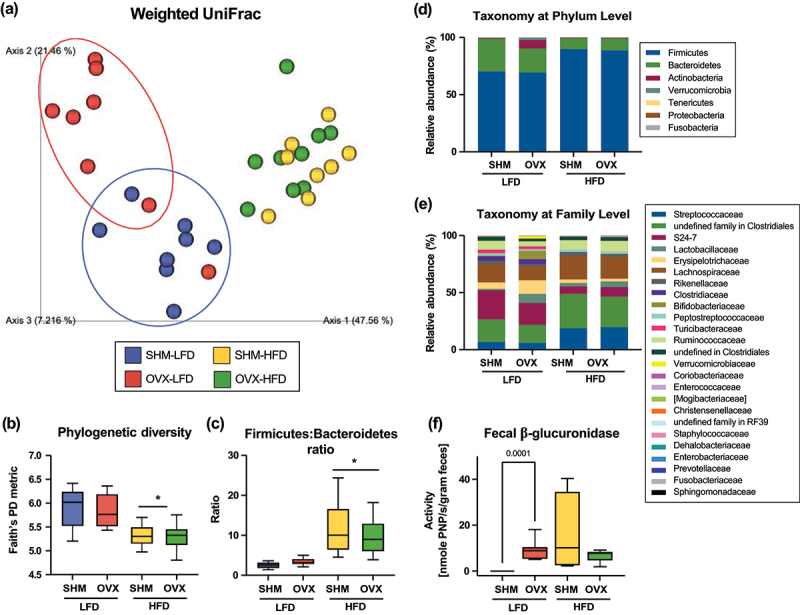
Cecal microbiota and fecal β-glucuronidase activity of ovariectomized (OVX) or sham-operated (SHM) conventionally raised C57BL/6J mice fed either a low-fat (LFD) or a high-fat diet (HFD). Weighted UniFrac principal coordinates analysis (PCoA) plots (a), phylogenetic diversity (b), Firmicutes:Bacteroidetes ratio (c), taxonomic classification at the phylum (d) and family (e) level of cecal microbiota, and the fecal β-glucuronidase activity (f) 12-week post ovariectomy intervention (*n*=8–10/group). *denotes main diet effect, *p* < .05.

Cecal contents were collected from mice at euthanasia to assess gut microbiota profiles. Principal coordinates analysis (PCoA) of weighted and unweighted UniFrac distances revealed a distinct separation (*p* < .05) of the cecal microbiota between HFD- and LFD-fed mice at the end of the intervention (Figure 3a). Interestingly, differential clustering (*p* < .05) was observed between SHM and OVX animals only when fed the LFD diet, but not among the HFD-fed groups. Our data suggest that diet exerts strong impacts on the gut microbiota, and the gut microbial differences due to sex hormone status may be masked by the HFD. Alpha diversity measures of species richness suggested that the HFD led to lower (*p* < .05) phylogenetic diversity than LFD (Figure 3b). However, species richness did not differ based on the sex hormone status between the OVX and SHM groups. We identified 7 phyla, 25 families, and 35 genera in the ceca of these mice. The most abundant phyla included Firmicutes (average 79.8% of sequences), Bacteroidetes (17.3%), Actinobacteria (2.2%), and Verrucomicrobia (0.6%). HFD promoted a greater Firmicutes:Bacteroidetes ratio (Figure 3c), which has been associated with an obese phenotype.^28^ We also observed distinct microbial profile of the OVX-LFD group relative to the rest of the treatment groups (Figure 3d,e). These mice have significantly greater proportion of Actinobacteria, driven by the genus *Bifidobacterium* within the family Bifidobacteriaceae (*p* < .05, Supplemental Table S5). The increase in *Bifidobacterium* observed in the gut microbiota of OVX-LFD mice was confirmed with quantitative PCR assessment (Supplemental Figure. S4). Despite the significant phenotypical differences observed between SHM and OVX mice fed the HFD, the gut microbiota did not differ between these two treatment groups. In contrast, when fed the LFD, differential clustering of the gut microbiota was observed between SHM and OVX mice.

Fecal samples were collected every 4 weeks to assess longitudinal microbiota changes due to the loss of ovarian hormone production and identify the time points when microbiota changes occur after removal of ovarian production of sex hormones (Supplemental Fig. S5). Similar to the cecal microbiota, diet exerted significant impacts with PCoA of weighted and unweighted UniFrac distances of fecal microbiota revealing a distinct separation (*p* < .05) between HFD- and LFD-fed mice at each timepoint. Throughout the course of this study, the fecal microbiota of mice fed the HFD did not cluster differentially based on ovarian hormone status. On the other hand, the loss of ovarian hormones shifted the fecal microbiota of mice fed the LFD. Shifting of the fecal microbiota between SHM-LFD and OVX-LFD appeared to start around 4 weeks post-ovariectomy and continue to evolve to cluster most distinctly at 12 weeks.

As mentioned above, bacteria encoding β-glucuronidase can deconjugate steroidal metabolites, including estrogen, and allow them to be reabsorbed.^11,12^ We quantified fecal β-glucuronidase activity 12-week post-surgery to determine whether OVX-induced microbiome changes were associated with changes in the ability to recycle steroidal compounds. We found that β-glucuronidase activity was greater in the LFD-OVX group than the LFD-SHM group (Figure 3f). Similar to our observations in the gut microbiota, differences in fecal β-glucuronidase activity due to the loss of ovarian hormones were only observed in the LFD-fed mice, but not those fed the HFD.

### Fecal microbiota transplantation of ovariectomy-associated microbiome led to greater weight gain and inflammatory signatures

**Figure 4.**
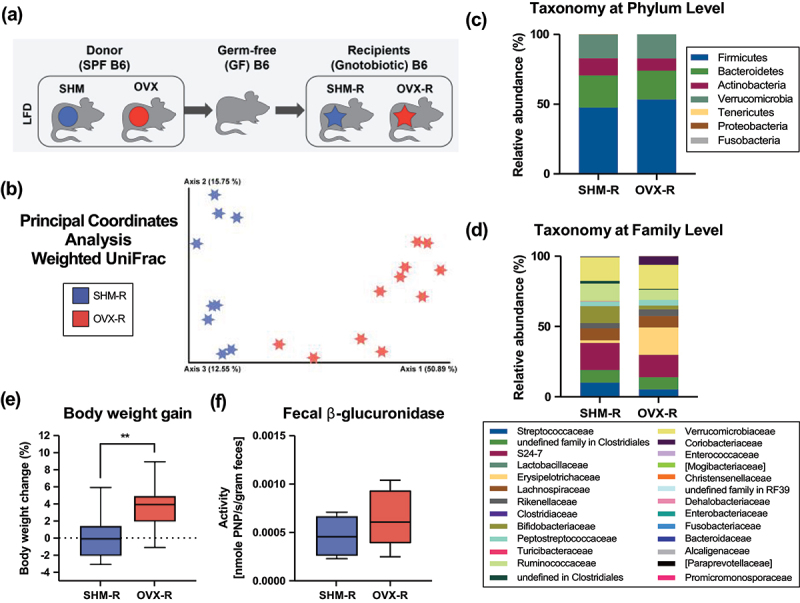
Gut microbiota and weight gain of gnotobiotic mice (R) colonized with fecal samples obtained from ovariectomized (OVX) or sham-operated (SHM) donor C57BL/6J mice fed a low-fat diet. Fecal microbiota transplantation (FMT) was performed by transplanting the gut microbiome from conventionally-raised OVX or SHM C57BL/6J (B6) mice fed the LFD into the germ-free (GF) recipients (a). The gut microbiota samples obtained from the HFD-fed donors were not used in the FMT study due the lack of distinct clustering of the gut microbiota based on treatment groups. Weighted UniFrac principal coordinates analysis (PCoA) plots (b), taxonomic classification at the phylum (c) and family (d) level of cecal microbiota, body weight gain (e) and the fecal β-glucuronidase activity (f) 4-week post-colonization at euthanasia (*n*=11–13/group).

Due to the lack of ovarian hormone-associated differences in the gut microbiota of HFD-fed mice, LFD-fed fecal samples from the aforementioned conventionally raised mice were used for the fecal microbiota transplant study (hereafter referred to as “donor” mice, Figure 4a). Fecal samples of the SHM and OVX donor mice fed the LFD were used to transplant into germ-free C57BL/6 mice to assess the metabolic phenotype induced by the gut microbiome. Fecal samples collected 8-week post-surgery from conventionally-raised donor mice were resuspended in rich media within an anaerobic chamber and used for inoculation. Germ-free recipient mice were fed an irradiated LFD with the same dietary formulation as conventionally raised donor mice and inoculated at 8 weeks of age. Four weeks after colonization, the cecal microbiota of the gnotobiotic recipient mice that received the SHM-associated microbiota (SHM-R) differentially clustered (*p* < .05) from those that received the OVX-associated microbiota (OVX-R) (Figure 4b). At the phylum level, OVX-R mice had lower relative abundance of Tenericutes and Proteobacteria compared to SHM-R-Mice (Figure 4c, Supplemental Table S6). At the family level, OVX-R mice had significantly greater (*p* < .05) relative abundance of *Erysipelotrichaceae*, *Coriobacteriaceae*, and *Clostridiaceae* as well as lower (*p* < .05) relative abundance of *Streptococcaceae*, *Enterobacteriaceae*, *Dehalobacteriaceae*, and *Bifidobacteriaceae* (Figure 4d, Supplemental Table S6). At the genus level, *Allobaculum* was significantly greater in OVX-R mice whereas *Bifidobacterium* and *Coprococcus* were significantly lower compared to SHM-R mice.

**Figure 5.**
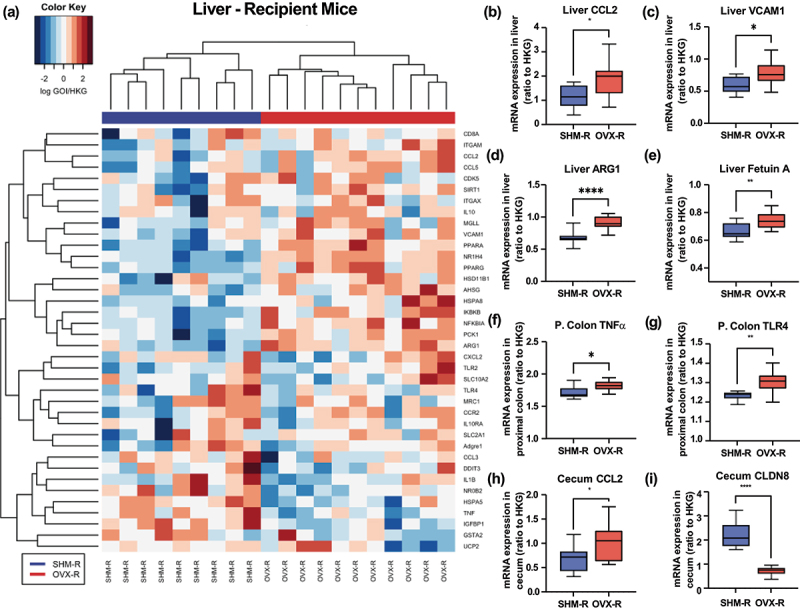
Gene expression of gnotobiotic recipient mice (R) four weeks after being colonized with microbiota of ovariectomized (OVX) or sham-operated (SHM) donor C57BL/6J mice fed a low-fat diet. Hierarchical clustering based on similarity of the hepatic gene expression (a) and relative expression of genes related to inflammation and tight junction proteins in liver, proximal colon (P. Colon), and cecum (b-i) of gnotobiotic mice. Each row of the heatmap represents a specific gene of interest, and each column represents one sample with color code denoting treatment groups (SHM-R: blue; OVX-R: red). *p < .05, **p < .01, ****p < .0001.

Four weeks after colonization, mice colonized with OVX-associated microbiota gained more (*p* < .05) weight than those receiving the SHM-associated microbiota (Figure 4e). Unlike donor mice, FMT did not lead to differences in the fecal β-glucuronidase activity between OVX-R and SHM-R mice 4-week post transplantation (Figure 4f). However, OVX-R mice had numerically greater fecal β-glucuronidase activity despite not statistically significant. Notably, the overall activity level detected in the recipient mice was markedly lower than the donor mice. Similar to donor mice, gene markers associated with inflammation, macrophages, oxidative stress, and hypoxia were assessed in the liver and intestines of the recipient mice. Hierarchical clustering strategy revealed a clear pattern similarity within each treatment group and dissimilarity between the groups based on the microbiota received, particularly in the liver (Figure 5a). Compared to SHM-R, mice received OVX-associated microbiota had greater (*p* < .05) hepatic expression of pro-inflammatory marker CCL2 (MCP1) and vascular cell adhesion molecule-1 (VCAM1) (Figure 5b,c). Upregulation (*p* < .05) of hepatic arginase I (ARG1) was also observed in the OVX-R group compared to that of SHM-R (Figure 5d). Greater expression of hepatic ARG1 has been associated with reduction in circulating nitric oxide level, which might lead to endothelial dysfunction.^29^ Interestingly, compared to SHM-R, OVX-R mice display a higher (*p* < .05) expression of hepatic fetuin A, a marker that has been associate with obesity, type 2 diabetes, metabolic syndrome, and nonalcoholic fatty liver disease (Figure 5e). OVX-R mice had greater (*p* < .05) expression of pro-inflammatory markers TNFα and toll-like receptors 4 (TLR4) in the proximal colon and CCL2 in the cecum compared to SHM-R (Figure 5f–h). Lower (*p* < .05) expression of tight junction protein encoding gene CLDN8 was observed in OVX-R mice, which may suggest a potential decrease in the intestinal barrier function in response to OVX-associated microbiota transplantation (Figure 5i).

## Discussion

Menopause-related loss of ovarian hormone production has been associated with increased risks of obesity and metabolic dysfunction. Obesity has been linked to disrupted gastrointestinal barrier function and shifts in the gut microbiome, while the obesity-associated microbiome alone is sufficient to promote metabolic dysfunction. Some evidence has suggested that menopause in humans is linked to changes in the gut microbiota.^30,31^ However, with menopause being an age-related loss of function, age is inevitably a confounding factor when examining the impact of menopause on the gut microbiome in humans. While comparisons with age-matched men account for the age variable, inherent biological differences based on sex and gender present other challenging confounders that warrant attention. Nonetheless, a recent report showed that the gut microbiota differences between pre-menopausal women and age-matched men were greater than the differences observed between post-menopausal women and age-matched men, implying the link exists between the menopause-associated loss of female sex hormones and the gut microbiota.^32^ Animal model of human menopause can be useful to provide insights in the relationship between female sex hormones and the gut microbiota while accounting for some of the inherent biases in humans. Previous research has demonstrated alterations in the gut microbiota in response to loss of ovarian hormones via ovariectomy in rodent models.^16,17,20^ However, the impact of these gut microbiome shifts on the development of the metabolic phenotype and inflammation remains unclear. Herein, we first utilized a diet-induced obesity approach in an ovariectomized female mouse model to examine the metabolic and inflammatory changes and the gut microbiome (bacterial membership present and enzyme activity) associated with ovariectomy. Further, we transplanted the gut microbiome of intact and ovariectomized mice fed a semi-purified LFD into germ-free mice to determine the contribution of the ovariectomy-associated gut microbiome on metabolic phenotypes. We demonstrated that ovariectomy led to greater gastrointestinal permeability and inflammation of the gut and metabolic organs, and that the obesity-promoting HFD exacerbated these conditions. Ovariectomy also led to alteration of the gut microbiota and greater fecal β-glucuronidase activity. However, these differential changes of the gut microbiome were only apparent when mice were fed the LFD, not the HFD. Hence, we utilized samples collected from OVX and SHM donor mice that were fed the LFD for the fecal microbiota transplantation experiment despite the metabolic phenotypes being more severe in those fed the HFD. Fecal microbiota transplantation demonstrated that the recipients of the OVX-associated gut microbiome had greater weight gain and hepatic gene expression related to inflammation, obesity, and atherosclerosis compared to those that received the SHM-associated microbiome. These results indicate that the gut microbiome responds to alteration in female sex hormone status and exacerbates metabolic dysfunction.

In the current study, a semi-purified diet free of phytoestrogens was utilized to feed both donor and recipient mice to eliminate this potential confounding dietary factor to impact estrogen signaling. Interestingly, we did not observe a community level impact of the gut microbiota due to ovariectomy in mice fed the high-sucrose semi-purified HFD. However, a much clearer differential clustering due to ovariectomy was evident in those fed the semi-purified LFD. Our observation contradicts that of previous findings that ovariectomy leads to distinct gut microbiota composition when fed a high-sucrose semi-purified HFD in Sprague-Dawley rats and two stains of mice (C57BL/6J and DBA/2J) compared to intact controls.^16,17^ The level of inclusion and sources of fat in the diets differed between our study and previous reports, with our study including a higher proportion of dietary fat and using lard as the main source (compared to milk fat used by Org et al.^16^, suggesting that dietary components may mask the gut microbiota alterations caused by changes in female sex hormones. Other studies using natural ingredient grain-based rodent chow have reported the lack of community level changes due to ovariectomy, including one of our own reports, while Cox-York and colleagues observed phylum-level gut microbial changes due to ovariectomy in 44-week-old low-capacity running (LCR) rats feeding a different natural ingredient-based diet.^16,18–20,33^ The impact of loss of ovarian production of sex hormone on the gut microbiota is likely highly sensitive to various factors, including diet fed, baseline microbiota, age, and host species and strains. Natural ingredient diets can contain inconsistent concentration of phytoestrogens due to varying inclusion amounts of soybean meal among production batches and may potentially bind to estrogen receptors within the gastrointestinal tract and elicit estrogen-like effects. Thus, dietary components and compositions should be carefully considered while dissecting the link between female sex hormones and the gut microbiome.

Female sex hormones have been shown to enhance gastrointestinal integrity, alter gastrointestinal transit time, and protect against intestinal injury, metabolic dysfunction, and inflammation.^34–36^ Therefore, loss of ovarian production of female sex hormone during menopause can compromise gastrointestinal function and significantly increase the risks of inflammatory and metabolic dysfunction in women. In the current study, gnotobiotic mice that received the gut microbiome from ovariectomy-associated donor mice (i.e., OVX-R group) had distinct microbiota profiles from those that received sham-associated microbiota (i.e., SHM-R group). Greater weight gain, hepatic and intestinal inflammatory gene expression, as well as lower intestinal tight junction protein-related gene expression were observed in the OVX-R mice compare to the SHM-R mice. Previous research has demonstrated that diet-induced and genetic-induced obesity-associated microbiota were able to increase energy harvest and body fat gain in gnotobiotic recipient mice^37^. However, whether the microbiota associated with ovariectomy-induced obesity from donors without extreme metabolic phenotypes can impact the metabolic phenotype of the hosts has not been demonstrated. A recent co-housing study exposing ovariectomized mice with “healthy” microbiota from lean, sham-operated controls failed to rescue the metabolic phenotype of mice with loss of ovarian hormone production.^20^ These authors then concluded that the gut microbiome likely does not contribute to the metabolic dysregulation associated with ovariectomy. However, surgical removal of ovaries causes drastic and powerful changes in sex hormone concentration, which leads to significant metabolic changes. The effects of the gut microbiome may not be as strong as a surgical cessation of sex hormones itself on host physiology, but its impact should not be neglected according to our study using unaltered mice as recipients. Fecal β-glucuronidase activity was assessed in this study due to its importance in maintaining sex hormone homeostasis through enterohepatic recycling of estrogens.^38^ In humans, fecal β-glucuronidase activity has been inversely correlated with fecal estrogens.^39^ In our current study, loss of ovarian hormones led to an increase in fecal β-glucuronidase in donor mice. Similar to the donor mice, OVX-R mice had numerically greater fecal β-glucuronidase activity, but not statistically significant. Notably, the detected level of β-glucuronidase activity in the feces of recipient mice was much lower than that of donor mice. It is possible that the length of colonization or priming window at birth affects activity level of gut microbial β-glucuronidase.

In our study, loss of ovarian production of female sex hormone and HFD feeding both lowered the expression of tight junction protein-related genes in donor mice when compared to sham intact controls and LFD, respectively. When transplanting the gut microbiome into gnotobiotic recipient mice, the expression of tight junction protein-related gene CLDN8 significantly decreased in OVX-R mice compared to the SHM-R group. We also observed that OVX-R mice had greater hepatic ARG1 expression compared to SHM-R. The ARG1 gene encodes enzyme arginase, which reduces nitric oxide production and bioavailability by competing with nitric oxide synthase for L-arginine and increases oxidative stress.^40^ Hepatic arginase can be released into circulation in the event of pathogen-induced liver damage, which can lead to plasma arginine depletion.^41^ Most of the total arginase activity in our body is contributed by hepatic arginase, which plays a critical role in eliminating nitrogenous waste produced through the urea cycle. Upregulation of arginase activity has been associated with multiple cardiovascular dysfunctions, including atherosclerosis and hypertension.^40,42^ An alteration of hepatic arginase from direct microbiome modulation has not been reported previously; however, arginase has been proposed to be a potential target in the treatment of cardiovascular diseases.^42^ Greater expression of hepatic fetuin-A and colonic TLR4 was also observed in OVX-R mice compared to SHM-R mice, suggesting an interaction between the gut microbiome and fetuin-A expression associated with altered sex hormone status. Fetuin-A, also known as alpha-2-HS glycoprotein (AHSG), is a plasma glycoprotein synthesized predominantly by hepatocytes in adults that promotes bone mineralization.^43,44^ Elevated fetuin-A is linked with lower bone mineral density, greater cardiovascular disease risks, and impairment of insulin signaling.^45–47^ HFD feeding has been shown to increase hepatic fetuin-A protein level and circulating fetuin-A protein concentration in rodents.^48,49^ Fetuin-A is thought to be an endogenous ligand of TLR4 that can mediate downstream NFκB pathways to induce inflammation and insulin resistance.^50,51^ Dietary supplementation of *Lactobacillus casei* has been shown to decrease circulating fetuin-A concentration in Type 2 diabetes patients and improve glycemic response.^52^ Administration of lactic acid-producing probiotics in HepG2 hepatocarcinoma cells improve fatty acid-induced insulin resistance through modulation of the fetuin-A/TLR4-JNK-NF-κB pathway.^53^ Our data suggest that the ovariectomy-induced impairment in gastrointestinal integrity may be mediated through the gut microbiome. Gene markers associated with cardiovascular dysfunction, insulin signaling, and obesity identified in our study may also be regulated by the gut microbiome. Determining the gut microbial modulators to regulate these genes may be useful in managing metabolic disorders related to the loss of ovarian hormone production of female sex hormones.

## Summary

Ovariectomy led to alterations in the gut microbiome and greater fecal β-glucuronidase activity, but these differential changes were only evident when mice were fed a low-fat diet. Fecal microbiota transplantation of the ovariectomy-associated microbiome resulted in greater weight gain and gene expression associated with metabolic dysfunction and inflammation in the recipient gnotobiotic mice compared to those that received the sham-associated microbiome. In summary, this work demonstrated that the gut microbiome may respond to host female sex hormone alterations and exacerbate metabolic dysfunction.

## Materials and methods

### Conventionally raised specific pathogen-free (SPF) mice husbandry

Forty 10-week-old female SPF C57BL/6J mice were purchased from Jackson Laboratory (Bar Harbor, ME) and randomly allotted to either a HFD (60% kcal from fat; D12492 formulation, except that soybean oil was replaced with corn oil, Research Diets, Inc., New Brunswick, NJ) or an LFD (10% kcal from fat; D12450J formulation, except that soybean oil was replaced with corn oil, Research Diets, Inc.) *ad libitum*. Formulation and analyzed chemical compositions of these two diets are shown in Supplemental Table S1. At 12-week-old, mice then underwent either OVX or SHM surgery. Briefly, a vertical incision was made through the skin above each side of the ovarian fat pad, followed by a horizontal incision through the muscle layer to expose the ovaries. Ovaries were either removed using a cauterizer (OVX group) or replaced back into the abdominal cavity (SHM group). Fecal samples were collected at baseline (week 0, prior to surgery), and 4-, 8-, and 12-week post-surgery. Fecal samples collected 8-week post-surgery were used as the donor microbiomes. Twelve weeks post-surgery, mice were euthanized via CO_2_ inhalation after a 4-h fast. Mice were housed individually in a temperature-controlled room with a 12-h light:12-h dark cycle. Animal care and study protocol were approved by the Institutional Animal Care and Use Committee at the University of Illinois Urbana-Champaign.

### Gnotobiotic mice husbandry

Twenty 6-week-old germ-free C57BL/6 mice were used as recipient animals and fed an irradiated LFD with the same formulation as conventionally raised donor mice described above. Fecal samples collected 8-week post-surgery from conventionally raised OVX and SHM mice (*n* = 7–9) fed LFD were resuspended in rich media within an anaerobic chamber and used to inoculate germ-free recipient mice. At 8-week-old, germ-free recipients were colonized with these fecal inoculants through fecal microbiota transplantation (FMT). Fecal inoculants were made by homogenizing fecal samples in 5 ml pre-reduced Mega Medium in an anaerobic chamber. Each recipient mice received 200ul of fecal inoculant via oral gavage. Recipient animals were group housed in an Allentown Sentry SPP sealed positive pressure caging system (Allentown Inc., Allentown, NJ) and weighed weekly. Mice were housed in a temperature-controlled room with a 12-h light: 12-h dark cycle. Tissues were collected 4-week post-colonization. Animal care and study protocol were approved by the University of Wisconsin-Madison Animal Care and Use Committee.

### Serum chemistry

Serum of the donor mice was profiled by measuring triglycerides, non-esterified fatty acids, alanine transaminase, aspartate aminotransferase, and alkaline phosphatase (Comparative Clinical Pathology Services; Columbia, MO).

### Hepatic steatosis

Hepatic steatosis of the donor mice was determined by histopathology and liver triglyceride analysis. Formalin-fixed, paraffin-embedded liver sections were sectioned and stained with hematoxylin and eosin (H&E) and the severity of hepatic steatosis was blindly evaluated by a veterinary pathologist at the University of Illinois. Liver triglyceride concentrations were determined as described previously.^54^ Briefly, liver samples were homogenized in 1 mL of lipid extraction solvent composed of 1:2 vol/vol methanol-chloroform and then gently agitated overnight at 4°C. An equal part of 4 mM MgCl was added, vortexed, and centrifuged for 1 h at 1,000 × g at 4°C. The organic phase was evaporated and reconstituted in 3:2 vol/vol butanol-Triton X-114 mix (Sigma, St. Louis, MO). Liver triglyceride concentrations then were determined using a commercially available kit (Sigma, St. Louis, MO) with a spectrophotometer (Synergy HT, BioTek, Winooski, VT).

### Adipocyte sizing

Formalin-fixed, paraffin-embedded subcutaneous and gonadal adipose tissues (SQAT and GDAT, respectively) of the donor mice were sectioned and stained with H&E. Images were taken using a Nanozoomer (C9600, Hamamatsu Photonics, Japan). The severity of adipose tissue steatosis was blindly evaluated by a pathologist at the University of Illinois. Adipocyte volume was calculated using the cross-sectional area obtained from perimeter tracings through ImageJ software (http://imagej.nih.gov/ij/).

### Intestinal permeability measurement

Gut barrier function was assessed ex vivo using a modified Ussing chamber system. Ussing chambers allow for precise assessment and comparison of the physiological intestinal permeability of intestines without influencing other potential confounding variables.^55^ Briefly, a section of cecum was resected, cut longitudinally, and mounted into modified Ussing chambers exposing 0.031 cm^2^ of the mucosal and serosal sides. After allowing 10–20 min to reach equilibrium, transmural resistance (Ohm x cm^2^) was measured.

### Lipopolysaccharide-binding protein (LPSBP) assay

Serum LPSBP was analyzed using a commercial kit (HK205–02 Mouse LBP ELISA Kit, Hycult Biotech Inc., Plymouth Meeting, PA).

### Gene expression

For donor mice, total RNA from SQAT and GDAT was isolated using RNeasy Lipid Tissue kits (Qiagen, Valencia, CA, USA). For both donor and recipient mice, total RNA from liver and intestines were isolated using RNeasy kits (Qiagen, Valencia, CA, USA). DNase digestion was performed using a RNase-Free DNase Set (Qiagen, Valencia, CA, USA). RNA concentration was determined using an ND-1000 spectrophotometer (Nanodrop Technologies, Wilmington, DE, USA). cDNA was synthesized using SuperScript III reverse transcriptase (Invitrogen, Carlsbad, CA, USA). Assays for specific gene expressions were analyzed using Fluidigm Microfluidics Dynamic Arrays (Fluidigm Corporation, South San Francisco, CA) at the Functional Genomic Unit of the Roy J. Carver Biotechnology Center at the University of Illinois. The list of genes assessed and primers used is shown in Supplemental Table S3 and S4. Genes ACTB, RPS13, and PPIA were used as housekeeping genes. Briefly, primers designed by and purchased from Fluidigm Deltagene Assays were pooled at 1 uL per assay for specific target amplifications using Taqman PreAmp Master Mix (Thermo Fisher, San Jose, CA). The thermal protocol of this step was 10 min of initial denaturation at 95°C, followed by 13–14 amplification cycles of 15 s at 95°C and 4 min at 60°C. Exonuclease treatment then was used to remove excess primers using Exonuclease I (New England BioLabs, Beverly, MA). Evagreen Supermix (BioRad Laboratories, Hercules, CA) was used for real-time amplification and detection under the following thermal protocol: 40 min at 70°C, 30s at 60°C, and 1 min at 95°C, followed by 30 amplification cycles of 5s at 96°C and 20s at 60°C. A dissociation curve for each primer was produced with a programmed temperature ramp from 60–95°C in 2 min, from which the melting temperature (T_m_) was calculated. Analysis was performed using Fluidigm Real-Time PCR analysis Software 4.1.3.

### Bacterial community characterization using sequencing-based technique

Total DNA from cecal contents and fecal pellets (*n* = 8–10/group) were extracted using Mo-Bio PowerSoil kits (MO BIO Laboratories, Inc., Carlsbad, CA). Concentration of extracted genomic DNA was quantified using a Qubit® 3.0 Fluorometer (Life Technologies, Grand Island, NY). 16S rRNA gene amplicons were generated using a Fluidigm Access Array (Fluidigm Corporation, South San Francisco, CA) in combination with Roche High Fidelity Fast Start Kit (Roche, Indianapolis, IN). The primers 515F (5’-GTGYCAGCMGCCGCGGTAA-3’) and 806 R

(5’-GGACTACNVGGGTWTCTAAT-3’) that target a 252 bp-fragment of V4 region were used for amplification (primers synthesized by IDT Corp., Coralville, IA).^56^ CS1 forward tag and CS2 reverse tag were added according to the Fluidigm protocol. Barcode was added to each DNA amplicon to distinguish individual samples. Quality of the amplicons was assessed using a Fragment Analyzer (Advanced Analytics, Ames, IA) to confirm amplicon regions and sizes. A DNA pool was generated by combining equimolar amounts of the amplicons from each sample. The pooled samples were then size selected on a 2% agarose E-gel (Life technologies, Grand Island, NY) and extracted using a Qiagen gel purification kit (Qiagen, Valencia, CA). Cleaned size-selected pooled products were run on an Agilent Bioanalyzer to confirm appropriate profile and average size. Illumina sequencing was performed on a MiSeq using 2 × 300v3 reagents (Illumina Inc., San Diego, CA). Library preparation and sequencing were performed at the Roy J. Carver Biotechnology Center at the University of Illinois. Forward reads were trimmed using the FASTX-Toolkit (version 0.0.13), and qiime2–2021.4 was used to process the resulting sequence data.^57^ Briefly, sequences were imported using the EMP protocol and denoised using DADA2^58^ to obtain the amplicon sequence variant (ASV) table. Singletons (ASV that were observed fewer than 2 times) and ASV that were present in less than 10% of the samples were discarded. A naive Bayes taxonomy classifier was trained on the Greengenes 13_8 reference database with a 99% similarity threshold.^59^ This classifier was used to assign taxonomy to ASV.^60^ For cecal samples of donors and recipients: an even sampling depth of 19,482 sequences per sample was used for assessing alpha- and beta-diversity measures. For longitudinal fecal samples of the donors collected at 0-, 4-, 8-, and 12-week post-surgery, an even sampling depth of 11,376 sequences per sample was used for assessing alpha- and beta-diversity measures.

### Specific bacterial taxa assessment using quantitative PCR

Quantitative PCR (qPCR) was performed as previously described^61,62^ using a Bio-Rad CFX384 real-time PCR detection system (Bio-Rad Laboratories, Inc., Hercules, CA) to quantitatively assess specific bacterial taxa in the cecal content of conventionally raised mice. Sequences of primers targeting all bacteria (universal primer), specific phyla (Bacteroidetes and Firmicutes), specific family (Ruminococcaceae), and specific genera (*Lactobacillus*, *Bifidobacterium*, *Blautia*, *Faecalibacterium*, *Fusobacterium*, *Turicibacter*, *Streptococcus*, *Enterococcus*) used are listed in Supplemental Table S5. qPCR data are expressed as the log DNA for each bacterial taxon/5 ng of isolated total DNA.

### Fecal β-glucuronidase activity

Frozen fecal samples collected from the donors 12-week post-surgery were first weighed and rehydrated in 15× weight/volume assay buffer (100 mM HEPES, 250 mM NaCl, pH 7.4). Bacterial cells were lysed using a Tissuelyzer II (Qiagen, Valencia, CA, USA) for two min at 30 Hz. Homogenates were sonicated for 3 min, then clarified by centrifugation at room temperature for 5 min at 13,000 × g. Enzyme kinetics were determined by monitoring the change in absorbance of p-nitrophenyl glucuronide (PNPG), a synthetic substrate. Reactions were conducted in quadruplicate in 96-well clear bottom assay plates (Corning Costar®, Thermo Fisher, San Jose, CA). The 50 μL reaction volume consisted of 10 μL clarified fecal homogenate, 10 μL assay buffer and 30 μL PNPG (final concentration 1 mM). Appropriate blanks and controls were included. All experimental manipulation until this point occurred at 4°C. Enzyme activity was measured for 1 h at 37°C using a PHERAstar Plus microplate reader (BMG Labtech, Cary, NC). Initial velocity of each sample was calculated using custom MATLAB scripts, which was then normalized to the original weight of the fecal sample, and represented as nmol of PNP catalyzed per second per gram of feces using the extinction coefficient of PNP and path length of the assay well.^63,64^

### Statistical analysis

Phenotypic data were analyzed using the MIXED procedure of SAS 9.3 (SAS Institute, Cary, NC) with diet and surgery being fixed effects and animal being a random effect when parametric analysis was appropriate. When a main effect was significant, post hoc pairwise comparisons were performed using Tukey’s multiple comparison tests. Data normality was checked using the UNIVARIATE procedure and Shapiro–Wilk statistic. Data are reported as means ± SEM with statistical significance set as *p* < .05 and *p* < .10 considered as trends. To evaluate the changes of genes assessed within GDAT, SQAT, and liver, heat maps were generated using gplots package in R. The relative concentration of each data point was calculated based on the dilution curves generated for each gene and all data points then underwent a Log2 transformation. Gene expression was normalized using the mean of housekeeping genes (ACTB, PPIA, and RPS13). For the microbiota data, ANCOMBC (v 2.2.0) was used to analyzed differential abundance of the ASV and taxonomy detected. Briefly, data obtained from QIIME2 was imported into R (v 2023.06.0 + 421) with the packages qiime2R (v 0.99.6) and phyloseq (v 1.44.0).^65^ Differentially abundant taxa among treatment groups at phylum, family, genus, species, and ASV levels were identified using Analysis of Composition of Microbiomes with Bias Correction 2 (ANCOM-BC2) from the R package ANCOMBC (v 2.2.0)^66^ using the default parameters, except that multiple test corrections were performed using the Benjamini–Hochberg procedure.

## Highlights

- Loss of ovarian production of female sex hormones impair gut integrity, especially when fed a high-fat diet
- Gut microbiome changes due to ovarian hormones only occur when fed a low-fat diet
- The gut microbiome responds to ovariectomy and exacerbates metabolic dysfunction

## Supplementary Material

Ovx_microbiome_Cross_GutMicrobes_Submission_SuppTables1_4.docx

SupplementalFig1.docx

Ovx_microbiome_Cross_GutMicrobes_Submission_SuppTables5_6.xlsx

SupplementalFig5.docx

SupplementalFig2.docx

SupplementalFig4.docx

SupplementalFig3.docx

## Data Availability

The raw sequences that support the findings of this study are available at the NCBI sequences read archive at https://www.ncbi.nlm.nih.gov/sra/, reference number BioProject ID PRJNA994490.
