# Supplementary material for: Gut microbiome responds to alteration in female sex hormone status and exacerbates metabolic dysfunction
Source: Gut Microbes. 2023 Dec 28;16(1):2295429. doi: 10.1080/19490976.2023.2295429 (PMC10761013; doi:10.1080/19490976.2023.2295429)
Supplement: Ovx_microbiome_Cross_GutMicrobes_Submission_SuppTables1_4.docx [file KGMI_A_2295429_SM1879.docx]

# Supplemental Table 1. Ingredient and analyzed chemical composition of the high-fat (HFD) and low-fat diets (LFD) fed to conventionally-raised mice

|  | **LFD** | **HFD** |
| --- | --- | --- |
| ***Ingredient*** | **g/kg diet (as-fed basis)** | |
| Casein, 80 mesh | 189.6 | 258.5 |
| L-cystine | 2.8 | 3.9 |
| Cornstarch | 479.8 | 0.0 |
| Maltodextrin 10 | 118.5 | 161.5 |
| Sucrose | 65.2 | 88.9 |
| Cellulose, Solka floc | 47.4 | 64.6 |
| Corn oil | 23.7 | 32.3 |
| Lard | 19.0 | 316.6 |
| Mineral mix^1^ | 9.5 | 12.9 |
| Dicalcium phosphate | 12.3 | 16.8 |
| Calcium carbonate | 5.2 | 7.1 |
| Potassium citrate | 15.6 | 21.3 |
| Vitamin mix^2^ | 9.5 | 12.9 |
| Choline bitartrate | 1.9 | 2.6 |
| ***Analyzed chemical composition*** |  | |
| Dry matter (DM, %) | 90.2 | 93.3 |
|  | --DM basis-- | |
| Organic matter (%) | 96.6 | 95.4 |
| Ash (%) | 3.4 | 4.6 |
| Crude protein (%) | 18.2 | 24.1 |
| Acid-hydrolyzed fat (%) | 7.8 | 39.5 |
| Nitrogen-free extract (%) | 65.5 | 25.8 |
| Total dietary fiber (%) | 5.1 | 6.1 |
| Gross energy (kcal/g diet) | 4.5 | 6.3 |
| Calculated metabolizable energy (Atwater factors, kcal/g DM) | 4.1 | 5.5 |

^1^ Each kg of mineral mix consists of: ammonium molybdate tetrahydrate (0.3 g); copper carbonate (1.05 g); ferric citrate (21 g); magnesium sulfate heptahydrate (257.6 g); manganese carbonate hydrate (12.25 g); chromium potassium sulfate (1.93 g); potassium iodate (0.04 g); sodium chloride (259 g); sodium fluoride (0.2 g); sodium selenite (0.04 g); zinc carbonate (5.6 g); magnesium oxide (41.9 g).

^2^Each kg of vitamin mix consists of: biotin, 1% (2 g); calcium pantothenate (1.6 g); folic acid (0.2 g); Vitamin K as menadione sodium bisulfite (0.08 g); niacin (3 g); pyridoxine-HCl (0.7 g); riboflavin (0.6 g); thiamine-HCl (0.6 g); vitamin A acetate (0.8 g, 500,000 IU/g); vitamin B12; 0.1% mannitol (1 g); vitamin D3 (1 g, 100,000 IU/g); vitamin E acetate, 50% (10 g).

Supplemental Table 2. Histopathology scoring of liver and adipose tissue^1^

|  | LFD | |  | HFD | |  | *p* values | | |
| --- | --- | --- | --- | --- | --- | --- | --- | --- | --- |
| Tissue | SHM | OVX |  | SHM | OVX |  | Diet | Surgery | Diet*Surgery |
| Liver^2^ | 0.44 ± 0.33 | 1.56 ± 0.35 |  | 0.38 ± 0.35 | 1.89 ± 0.33 |  | 0.71 | <0.01 | 0.57 |
| GDAT^3^ | 0.44 ± 0.23 | 0.38 ± 0.25 |  | 1.63 ± 0.25 | 2.11 ± 0.23 |  | <0.01 | 0.40 | 0.26 |

^1^ Values are least-squares means; n=8-10. LFD: low-fat diet; HFD: high-fat diet; SHM: sham-operated; OVX: ovariectomized; GDAT: gonadal adipose tissue. Interactions were denoted by superscript letters, with means lacking a common letter within each tissue being different (*p*<0.05).

^2^ Hepatic steatosis/lipidosis based on a severity scale of 0-5 (0, normal; 1, minimal; 2, mild; 3, moderate; 4, marked; 5, severe; 6, very severe).

^3^ Adipose tissue steatitis based on a severity scale of 0-5 (0, normal; 1, minimal, focal or multifocal interstitial aggregates of 3-5 histiocytes/lymphocytes involving less than 25% of the area of adipose on the slide; 2, mild, multifocal interstitial aggregates of 3-5 histiocytes/lymphocytes involving more than 25% of the area of adipose on the slide; 3, moderate, multifocal interstitial aggregates of 5-10 histiocytes/lymphocytes involving less than 25% of the adipose tissue on the slide; 4, marked, multifocal to locally extensive interstitial aggregates of 5-10 histiocytes/lymphocytes involving 25-50% of the adipose on the slide; 5, severe, multifocal to locally extensive interstitial aggregates of >10 histiocytes/lymphocytes involving >50% of the adipose on the slide.

# Supplemental Table 3. Primer sequences of genes assessed in gonadal and subcutaneous adipose tissue and liver of C57BL/6 mice using qRT-PCR

| **Target** | **Forward Primer** | **Reverse Primer** | **Gene Full Name** | **Gene Aliases/**  **Target Protein** |
| --- | --- | --- | --- | --- |
| **ACACA** | GACAACACCTGTGTGGTGGAA | TGTTTAGCGTGGGGATGTTCC | acetyl-Coenzyme A carboxylase alpha | Acc1 |
| **ACTB** | CCCTAAGGCCAACCGTGAAA | AGCCTGGATGGCTACGTACA | actin, beta | Beta-actin |
| **Adgre1** | GCAGAAGCTCTGCAGTGTCA | AATCTGGGCAATGGCCTTGAA | adhesion G protein-coupled receptor E1 | F4/80 |
| **ADIPOQ** | TCCTGGAGAGAAGGGAGAGAA | TCAGCTCCTGTCATTCCAACA | adiponectin, C1Q and collagen domain containing |  |
| **AHSG** | GTGGCCTGCAAGTTATTCCAA | CTGCATTCGCTGTGGGTAC | alpha-2-HS-glycoprotein | Fetuin-A |
| **ANGPT2** | GAACCAGACAGCAGCACAAA | TCGAGTCTTGTCGTCTGGTTTA | angiopoietin 2 |  |
| **ARG1** | GGATTGGCAAGGTGATGGAA | CGACATCAAAGCTCAGGTGAA | arginase, liver |  |
| **BCL2** | ATGTGTGTGGAGAGCGTCAA | GATGCCGGTTCAGGTACTCA | B cell leukemia/lymphoma 2 |  |
| **CASP3** | AGTCTGACTGGAAAGCCGAAA | TCTGTCTCAATGCCACAGTCC | caspase 3 |  |
| **CAT** | GGGATCTTGTGGGAAACAACAC | CTGTGGGTTTCTCTTCTGGCTA | catalase | Cas1 |
| **CCL2** | AGCAGCAGGTGTCCCAAA | TTCTTGGGGTCAGCACAGAC | chemokine (C-C motif) ligand 2 | Mcp1 |
| **CCL3** | ACCATGACACTCTGCAACCA | GAATCTTCCGGCTGTAGGAGAA | chemokine (C-C motif) ligand 3 |  |
| **CCL5** | GTGCCCACGTCAAGGAGTA | GCGGTTCCTTCGAGTGACA | chemokine (C-C motif) ligand 5 |  |
| **CCR2** | TGAGGCTCATCTTTGCCATCA | GGATTCCTGGAAGGTGGTCAA | chemokine (C-C motif) receptor 2 |  |
| **CCR5** | TAGCCAGAGGAGGTGAGACA | CGGAACTGACCCTTGAAAATCC | chemokine (C-C motif) receptor 5 | AM4-7\|CD195 |
| **CD19** | CCATCGAGAGGCACGTGAA | ACCACTGGGACTATCCATCCA | CD19 antigen |  |
| **CD3E** | TGCTACACACCAGCCTCAAA | AGGTCCACCTCCACACAGTA | CD3 antigen, epsilon polypeptide |  |
| **CD4** | AAGGGACACTGCATCAGGAA | CCCATCACCTCACAGGTCAA | CD4 antigen |  |
| **CD68** | ATCCCCACCTGTCTCTCTCA | CTGTACTCGGGCTCTGATGTA | CD68 antigen |  |
| **CD8A** | CAGCAAGGAAAACGAAGGCTAC | GCAGCACTGGCTTGGTAGTA | CD8 antigen, alpha chain | Ly-2\|Ly-B\|Ly-35 |
| **CDK5** | CGTGCTACATAGGGACCTGAA | AGGCCAAAATCAGCCAATTTCA | cyclin-dependent kinase 5 | Crk6 |
| **CNR1** | ACAAGCACGCCAATAACACA | TGGTCACCTTGGCGATCTTA | cannabinoid receptor 1 (brain) | CB1\|CB-R\|CB1R |
| **CPT1A** | CTGCCTCTATGTGGTGTCCAA | ACAACCTCCATGGCTCAGAC | carnitine palmitoyltransferase 1a, liver | CPTI\|Cpt1 |
| **DGAT2** | TTGGCTACGTTGGCTGGTAA | GTCTATGGTGTCTCGGTTGACA | diacylglycerol O-acyltransferase 2 | ARAT\|DGAT-2 |
| **EPAS1** | AAGCTTTTCGCCATGGACAC | CAAGGTCTCCAAATCCAGTTCAC | endothelial PAS domain protein 1 | HIF2alpha |
| **FAAH** | TGGCCTGAAGAGCTGTGTTTA | ATGCCAGGCTATCCACATCC | fatty acid amide hydrolase | AW412498 |
| **FAS** | TGTTTTCCCTTGCTGCAGAC | CCGCCTCCTCAGCTTTAAAC | Fas (TNF receptor superfamily member 6) | CD95 |
| **FASL** | CGAGGAGTGTGGCCCATTTA | AGCGGTTCCATATGTGTCTTCC | Fas ligand (TNF superfamily, member 6) | CD95L |
| **FCGR1** | ATCTGCAGGAGTGTCCATCA | AGATGACACGGATGCTCTCA | Fc receptor, IgG, high affinity I | CD64\|IGGHAFC \|FcgammaRI |
| **GSTA2** | GCCTTGGCAAAAGACAGGAC | GTCTTGTCCATGGCTCTTCAAC | glutathione S-transferase, alpha 2 (Yc2) | Gstc2\|Gst2-2\|Gstc-2 |
| **HIF1A** | TCGACACAGCCTCGATATGAA | TTCCGGCTCATAACCCATCA | hypoxia inducible factor 1, alpha subunit | HIF1alpha |
| **HSD11B1** | AAAATGGGAGCCCATGTGGTA | AGTTCAAGGCAGCGAGACA | hydroxysteroid 11-beta dehydrogenase 1 |  |
| **ICAM1** | AGGGCTGGCATTGTTCTCTA | TGTCGAGCTTTGGGATGGTA | intercellular adhesion molecule 1 | CD54 |
| **IFNG** | CCACGGCACAGTCATTGAAA | GCCAGTTCCTCCAGATATCCAA | interferon gamma |  |
| **IGFBP1** | TCTGCCAAACTGCAACAAGAA | CCACTCCATGGGTAGACACA | insulin-like growth factor binding protein 1 |  |
| **IKBKB** | GTTCGCTACCCTTCCCCAATA | AGGGTGCCACATAAGCATCA | inhibitor of kappaB kinase beta | IKK2\|IKKbeta |
| **IL10** | AAAGGACCAGCTGGACAACA | TAAGGCTTGGCAACCCAAGTA | interleukin 10 |  |
| **IL10RA** | GGTCGGAGGAGCAGTGTTTA | AAGATGCTCAGGTTGGTCACA | interleukin 10 receptor, alpha |  |
| **IL1B** | TGGCAACTGTTCCTGAACTCA | GGGTCCGTCAACTTCAAAGAAC | interleukin 1 beta |  |
| **IL6** | CGATGATGCACTTGCAGAAA | ACTCCAGAAGACCAGAGGAA | interleukin 6 |  |
| **IRS1** | GCAGCCAGAGGATCGTCAATA | CGTGAGGTCCTGGTTGTGAA | insulin receptor substrate 1 | IRS-1 |
| **IRS2** | ACCTATGCAAGCATCGACTTCC | GGGCTGGTAGCGCTTCA | insulin receptor substrate 2 | Irs-2 |
| **ITGAM** | AGCAGCTGAATGGGAGGAC | GGCCCCATTGGTTTTGTGAA | integrin alpha M | CD11b |
| **ITGAX** | AAGGCAGCTAAGAGGGTACAC | TCCACTTTGGGTGGTGAACA | integrin alpha X | CD11c |
| **LEP** | AGACCATTGTCACCAGGATCA | ATGAAGTCCAAGCCAGTGAC | leptin | ob\|obese |
| **LIPE** | GCTACACAAAGGCTGCTTCTAC | TGGAGAGAGTCTGCAGGAAC | lipase, hormone sensitive | HSL |
| **LPL** | GAGAGCGAGAACATTCCCTTCA | CGATGTCCACCTCCGTGTAA | lipoprotein lipase | Lpl |
| **MGLL** | GTCAATGCAGACGGACAGTAC | CATAACGGCCACAGTGTTCC | monoglyceride lipase | Mgl\|Magl |
| **MRC1** | AAGGCTATCCTGGTGGAAGAA | CTCTCGTGAATTGCCACCAA | mannose receptor, C type 1 | CD206 |
| **NFKBIA** | GAGCGAGGATGAGGAGAGCTA | GGCCTCCAAACACACAGTCA | nuclear factor of kappa light polypeptide gene enhancer in B cells inhibitor, alpha | Nfkbi |
| **NOS2** | GAGGAGCAGGTGGAAGACTA | GGAAAAGACTGCACCGAAGATA | nitric oxide synthase 2, inducible | iNOS |
| **NR3C1** | CAAGTGATTGCCGCAGTGAA | AGAAGGGTCATTTGGTCATCCA | nuclear receptor subfamily 3, group C, member 1 | GR\|Grl1\|Grl-1 |
| **PCK1** | CCGTCTGGCTAAGGAGGAA | TCTTCTTGCCTTCGGGGTTA | phosphoenolpyruvate carboxykinase 1, cytosolic | PEPCK\|Pck-1 |
| **PLIN2** | AGGATGGAGGAAAGACTGCCTA | ACATCCTTCGCCCCAGTTAC | perilipin 2 | ADPH\|Adfp\|Adrp |
| **PPARA** | TTCCCTGTTTGTGGCTGCTA | CCTGCAACTTCTCAATGTAGCC | peroxisome proliferator activated receptor alpha | Ppar\|Nr1c1 \|PPARalpha |
| **PPARG** | ACCCAATGGTTGCTGATTACA | AGGTGGAGATGCAGGTTCTA | peroxisome proliferator activated receptor gamma |  |
| **PPARGC1A** | AAACCACACCCACAGGATCA | GCTCTTCGCTTTATTGCTCCA | peroxisome proliferative activated receptor, gamma, coactivator 1 alpha | PGC1alpha |
| **PPIA** | AGGGTTCCTCCTTTCACAGAA | TGCCGCCAGTGCCATTA | peptidylprolyl isomerase A |  |
| **RBP4** | CTGTGGACGAGAAGGGTCATA | CATGTCTGCACACACTTCCC | retinol binding protein 4, plasma |  |
| **RPS13** | TCCTGTGCGGCTTGATTTCC | GGTCTGGCAGCAAAGAGAGAA | ribosomal protein S13 | 2700063M04Rik |
| **SERPINE1** | CAGACAATGGAAGGGCAACA | GAGGTCCACTTCAGTCTCCA | serine (or cysteine) peptidase inhibitor, clade E, member 1 | Plasminogen Activator Inhibitor\|PAI-1 |
| **SFRP5** | GAACAGATGTGCTCCAGTGAC | CTTTCGGTCCCCGTTGTCTA | secreted frizzled-related sequence protein 5 | SARP3 |
| **SLC2A1** | GCTGTGCTGTGCTCATGAC | GATGGCCACGATGCTCAGATA | solute carrier family 2 (facilitated glucose transporter), member 1 | Glut1\|Glut-1 |
| **SLC2A4** | TGTCGGCATGGGTTTCCA | AAGCAGGAGGACGGCAAATA | solute carrier family 2 (facilitated glucose transporter), member 4 | Glut4\|Glut-4 |
| **SPP1** | TGCCTGACCCATCTCAGAA | AAGTCATCCTTTTCTTCAGAGGAC | secreted phosphoprotein 1 | Osteopontin\|Opn\|Ric\|BNSP\|BSPI\|Opnl\|Apl-1\|ETA-1\|Spp-1 |
| **SREBF1** | ACCCTACGAAGTGCACACAA | CACATCTGTGCCTCCTCCA | sterol regulatory element binding transcription factor 1 | ADD1\|SREBP1  \|bHLHd1\|SREBP1c  \|SREBP-1a |
| **TLR2** | TGCATCACCGGTCAGAAAAC | AGCCAAAGAGCTCGTAGCA | toll-like receptor 2 | Ly105 |
| **TLR4** | GTTCTTCTCCTGCCTGACAC | GCTGAGTTTCTGATCCATGCA | toll-like receptor 4 |  |
| **TNF** | GGGTGATCGGTCCCCAAA | TGAGGGTCTGGGCCATAGAA | tumor necrosis factor | TNFalpha |
| **UCP2** | GGTCACTGTGCCCTTACCA | ATCCCAAGCGGAGAAAGGAA | uncoupling protein 2 (mitochondrial, proton carrier) | Slc25a8 |
| **UCP3** | TGTGCTGAGATGGTGACCTA | GCTCCAAAGGCAGAGACAAA | uncoupling protein 3 (mitochondrial, proton carrier) | UCP-3\|Slc25a9 |
| **VCAM1** | CCCAAACAGAGGCAGAGTGTA | TGACCCAGATGGTGGTTTCC | vascular cell adhesion molecule 1 | CD106 |
| **VEGFA** | CCAGCACATAGGAGAGATGAG | CTGGCTTTGTTCTGTCTTTCTT | vascular endothelial growth factor A |  |

# Supplemental Table 4. Primer sequences of genes assessed in intestines of C57BL/6 mice using qRT-PCR

| **Target** | **Forward Primer** | **Reverse Primer** | **Gene Full Name** | **Gene Aliases/**  **Target Protein** |
| --- | --- | --- | --- | --- |
| **ACACA** | GACAACACCTGTGTGGTGGAA | TGTTTAGCGTGGGGATGTTCC | acetyl-Coenzyme A carboxylase alpha | Acc1 |
| **ACACB** | TTGGAGGCAACAGGGTCATA | ATGGAGCGCATACACTTGAC | acetyl-Coenzyme A carboxylase beta | Acc2 |
| **ACTB** | CCCTAAGGCCAACCGTGAAA | AGCCTGGATGGCTACGTACA | actin, beta | Beta-actin |
| **ANGPTL4** | CTTGGGACCAAGACCATGAC | TGGCTACAGGTACCAAACCA | angiopoietin-like 4 | Fiaf\|fasting-induced adipose factor |
| **APAF1** | CACAGACCTTTCCATCCTTCA | CGTTTCCAAGTCCCAGAGAA | apoptotic peptidase activating factor 1 | fog\|Apaf-1 |
| **BCL2** | ATGTGTGTGGAGAGCGTCAA | GATGCCGGTTCAGGTACTCA | B cell leukemia/lymphoma 2 |  |
| **CASP3** | AGTCTGACTGGAAAGCCGAAA | TCTGTCTCAATGCCACAGTCC | caspase 3 |  |
| **CAT** | GGGATCTTGTGGGAAACAACAC | CTGTGGGTTTCTCTTCTGGCTA | catalase | Cas1 |
| **CCL2** | AGCAGCAGGTGTCCCAAA | TTCTTGGGGTCAGCACAGAC | chemokine (C-C motif) ligand 2 | Mcp1 |
| **CCL3** | ACCATGACACTCTGCAACCA | GAATCTTCCGGCTGTAGGAGAA | chemokine (C-C motif) ligand 3 |  |
| **CCL5** | GTGCCCACGTCAAGGAGTA | GCGGTTCCTTCGAGTGACA | chemokine (C-C motif) ligand 5 |  |
| **CCR2** | TGAGGCTCATCTTTGCCATCA | GGATTCCTGGAAGGTGGTCAA | chemokine (C-C motif) receptor 2 |  |
| **CCR5** | TAGCCAGAGGAGGTGAGACA | CGGAACTGACCCTTGAAAATCC | chemokine (C-C motif) receptor 5 | AM4-7\|CD195 |
| **CLDN1** | GCCACAGCATGGTATGGAAAC | AGGGCCTGGCCAAATTCA | claudin 1 | AI596271 |
| **CLDN2** | TTCCAGAGCTCTTCGAAAGGAC | GTATCTGTGGGTGGCCACAA | claudin 2 | AL022813 |
| **CLDN3** | GTGTACCAACTGCGTACAAGAC | GCCAACAGGAAAAGCACTCC | claudin 3 | mRVP1\|Cpetr2 |
| **CLDN4** | CGTGGCAAGCATGCTGATTA | GGAAGCCACCATAGGGTTGTA | claudin 4 | Cep-r\|Cpetr\|Cpetr1 |
| **CLDN5** | GCTGGTGGCACTCTTTGTTA | AGTGCTACCCGTGCCTTAA | claudin 5 |  |
| **CLDN8** | TTGCTGACAGCCGGAATCA | GAATTGGCAACCCAGCTGAC | claudin 8 | AI648025 |
| **CXCL2** | CCCCTGGTTCAGAAAATCATCC | TCCTTTCCAGGTCAGTTAGCC | chemokine (C-X-C motif) ligand 2 | IL8 |
| **DDIT3** | GGGGCACCTATATCTCATCCC | CGCAGGGTCAAGAGTAGTGAA | DNA-damage inducible transcript 3 | chop\|CHOP10 |
| **FFAR2** | AAGAGCAGCTGGATGTGGTA | GACTGCCATGGGAACGAAAA | free fatty acid receptor 2 | Gpr43 |
| **FFAR3** | CAGAGTGCCAGTTGTCCAATAC | ACACCAACAGGTAGACGGAAA | free fatty acid receptor 3 | Gpr41 |
| **FGF15** | CGCGGACGGCAAGATATAC | ACAGTCCATTTCCTCCCTGAA | fibroblast growth factor 15 | FGF19 |
| **FOXO1** | GAAGAGCGTGCCCTACTTCA | GGACAGATTGTGGCGAATTGAA | forkhead box O1 |  |
| **HIF1A** | TCGACACAGCCTCGATATGAA | TTCCGGCTCATAACCCATCA | hypoxia inducible factor 1, alpha subunit | HIF1alpha |
| **HSPA5** | TGCTGAGGCGTATTTGGGAA | TCGCTGGGCATCATTGAAGTA | heat shock protein 5 | Bip\|Sez7\|mBiP\|Grp78\|SEZ-7\|Hsce70 |
| **HSPA8** | GCAGCTGGGCCTACACA | GTAGGTGGTGCCGAGATCAA | heat shock protein 8 | Hsc70\|Hsc71\|Hsc73\|Hsp73\|Hspa10 |
| **IFNG** | CCACGGCACAGTCATTGAAA | GCCAGTTCCTCCAGATATCCAA | interferon gamma |  |
| **IKBKB** | GTTCGCTACCCTTCCCCAATA | AGGGTGCCACATAAGCATCA | inhibitor of kappaB kinase beta | IKK2\|IKKbeta |
| **IL10** | AAAGGACCAGCTGGACAACA | TAAGGCTTGGCAACCCAAGTA | interleukin 10 |  |
| **IL10RA** | GGTCGGAGGAGCAGTGTTTA | AAGATGCTCAGGTTGGTCACA | interleukin 10 receptor, alpha |  |
| **IL1B** | TGGCAACTGTTCCTGAACTCA | GGGTCCGTCAACTTCAAAGAAC | interleukin 1 beta |  |
| **IL6** | CGATGATGCACTTGCAGAAA | ACTCCAGAAGACCAGAGGAA | interleukin 6 |  |
| **IRS1** | GCAGCCAGAGGATCGTCAATA | CGTGAGGTCCTGGTTGTGAA | insulin receptor substrate 1 | G972R\|IRS-1 |
| **IRS2** | ACCTATGCAAGCATCGACTTCC | GGGCTGGTAGCGCTTCA | insulin receptor substrate 2 | Irs-2 |
| **LEP** | AGACCATTGTCACCAGGATCA | ATGAAGTCCAAGCCAGTGAC | leptin | ob\|obese |
| **LIPE** | GCTACACAAAGGCTGCTTCTAC | TGGAGAGAGTCTGCAGGAAC | lipase, hormone sensitive | HSL |
| **LPL** | GAGAGCGAGAACATTCCCTTCA | CGATGTCCACCTCCGTGTAA | lipoprotein lipase | Lpl |
| **MUC2** | CAGCACACCAACCAAAACCA | CACAGCCACCAGGTCTCATTA | mucin 2 | MCM\|wnn |
| **NFKBIA** | GAGCGAGGATGAGGAGAGCTA | GGCCTCCAAACACACAGTCA | nuclear factor of kappa light polypeptide gene enhancer in B cells inhibitor, alpha | Nfkbi\|AI462015 |
| **NLRP3** | TGCTCTGCAACCTCCAGAAA | AACCAATGCGAGATCCTGACA | NLR family, pyrin domain containing 3 | FCU\|MWS\|FCAS\|Cias1\|Mmig1\|NALP3\|Pypaf1 |
| **NR0B2** | GAAGGGCACGATCCTCTTCAA | GGGCTCCAAGACTTCACACA | nuclear receptor subfamily 0, group B, member 2 | SHP\|Shp1\|SHP-1 |
| **NR1H4** | GCCTCTGGGTACCACTACAA | GTACACGGCGTTCTTGGTAA | nuclear receptor subfamily 1, group H, member 4 | Fxr\|HRR1\|RIP14  \|Rxrip14 |
| **OCLN** | ACTGGTCTCTACGTGGATCA | ACCCCAGGACAATGGCTATA | occludin | Ocl |
| **PNPLA2** | TGTCTGCAGCACATTTATCCC | GAAATGCCGCCATCCACATA | patatin-like phospholipase domain containing 2 | Atgl\|TTS-2.2 |
| **PPARA** | TTCCCTGTTTGTGGCTGCTA | CCTGCAACTTCTCAATGTAGCC | peroxisome proliferator activated receptor alpha | Ppar\|Nr1c1 \|PPARalpha |
| **PPARG** | ACCCAATGGTTGCTGATTACA | AGGTGGAGATGCAGGTTCTA | peroxisome proliferator activated receptor gamma |  |
| **PPARGC1A** | AAACCACACCCACAGGATCA | GCTCTTCGCTTTATTGCTCCA | peroxisome proliferative activated receptor, gamma, coactivator 1 alpha | PGC1alpha |
| **PPIA** | AGGGTTCCTCCTTTCACAGAA | TGCCGCCAGTGCCATTA | peptidylprolyl isomerase A |  |
| **PRKAA1** | ATGTCTCTGGAGGAGAGCTA | GGAAAGGATCTGCTGGAACA | protein kinase, AMP-activated, alpha 1 catalytic subunit | AMPKalpha1 |
| **PRKAA2** | CGGAGCTATCTTCTGGACTTCA | CGCTGGGGTGTTGAAGAAC | protein kinase, AMP-activated, alpha 2 catalytic subunit | AMPKalpha2 |
| **PRKAB1** | CTTGTCTGGGTCCTTCAACAAC | CAGGTCCAGGATGGCTACAA | protein kinase, AMP-activated, beta 1 non-catalytic subunit | AMPKbeta1 |
| **PRKAB2** | CCTTACTTCCTGAGCCCAATCA | TTGCGCTAAGGACCATCACA | protein kinase, AMP-activated, beta 2 non-catalytic subunit | AMPKbeta2 |
| **PYCARD** | GAGCAGCTGCAAACGACTAA | TGTACTCTGAGCAGGGACAC | PYD and CARD domain containing | Asc\|TNS1\|masc\|CARD5 |
| **RBP4** | CTGTGGACGAGAAGGGTCATA | CATGTCTGCACACACTTCCC | retinol binding protein 4, plasma |  |
| **RPS13** | TCCTGTGCGGCTTGATTTCC | GGTCTGGCAGCAAAGAGAGAA | ribosomal protein S13 | 2700063M04Rik |
| **SIRT1** | CTGAAAGTGAGACCAGTAGCA | GATGAGGCAAAGGTTCCCTA | sirtuin 1 | Sir2\|Sir2a\|SIR2L1 \|Sir2alpha |
| **SLC10A2** | TGGAGGAACTGGCTCCAATA | GGAGCAAGTGGTCATGCTAA | solute carrier family 10, member 2 | ASBT\|ISBT |
| **SLC51A** | TTGCCCCATCAAGAAGAGGAC | AGAGACCAAAGCAGCAGAACA | solute carrier family 51, alpha subunit | Osta\|OSTalpha |
| **SREBF1** | ACCCTACGAAGTGCACACAA | CACATCTGTGCCTCCTCCA | sterol regulatory element binding transcription factor 1 | ADD1\|SREBP1\|bHLHd1\|SREBP1c\|SREBP-1a |
| **TJP1** | TCTGGCATCATTCGCCTTCA | TCAACCGCATTTGGCGTTAC | tight junction protein 1 | Zo-1 |
| **TLR2** | TGCATCACCGGTCAGAAAAC | AGCCAAAGAGCTCGTAGCA | toll-like receptor 2 | Ly105 |
| **TLR4** | GTTCTTCTCCTGCCTGACAC | GCTGAGTTTCTGATCCATGCA | toll-like receptor 4 |  |
| **TNF** | GGGTGATCGGTCCCCAAA | TGAGGGTCTGGGCCATAGAA | tumor necrosis factor | TNFalpha |
| **UCP2** | GGTCACTGTGCCCTTACCA | ATCCCAAGCGGAGAAAGGAA | uncoupling protein 2 (mitochondrial, proton carrier) | Slc25a8 |
| **UCP3** | TGTGCTGAGATGGTGACCTA | GCTCCAAAGGCAGAGACAAA | uncoupling protein 3 (mitochondrial, proton carrier) | UCP-3\|Slc25a9 |

# Supplemental Table 5. Primer sequences of specific bacterial taxa assessed in cecal content using qPCR

| **Taxa** | **Forward primer** | **Reverse primer** |
| --- | --- | --- |
|  |  |  |
| Bacteroidetes | CCGGAWTYATTGGGTTTAAAGGG | GGTAAGGTTCCTCGCGTA |
| *Bifidobacterium* spp. | TCGCGTCYGGTGTGAAAG | CCACATCCAGCRTCCAC |
| *Blautia* spp | TCTGATGTGAAAGGCTGGGGCTTA | GGCTTAGCCACCCGACACCTA |
| *Enterococcus* | CCCTTATTGTTAGTTGCCATCATT | ACTCGTTGTACTTCCCATTGT |
| *Faecalibacterium* spp. | GAAGGCGGCCTACTGGGCAC | GTGCAGGCGAGTTGCAGCCT |
| Firmicutes | GGCAGCAGTRGGGAATCTTC | ACACYTAGYACTCATCGTTT |
| *Fusobacterium* spp. | KGGGCTCAACMCMGTATTGCGT | TCGCGTTAGCTTGGGCGCTG |
| *Rumincoccaceae* | ACTGAGAGGTTGAACGGCCA | CCTTTACACCCAGTAAWTCCGGA |
| *Streptococcus* spp. | TTATTTGAAAGGGGCAATTGCT | GTGAACTTTCCACTCTCACAC |
| *Lactobacillus* spp. | AGCAGTAGGGAATCTTCCA | CACCGCTACACATGGAG |
| *Turicibacter* spp. | CAGACGGGGACAACGATTGGA | TACGCATCGTCGCCTTGGTA |
| Universal 16S | CCTACGGGAGGCAGCAGT | ATTACCGCGGCTGCTGG |
