# Supplementary figures and images for: Gut microbiome responds to alteration in female sex hormone status and exacerbates metabolic dysfunction
Source: Gut Microbes. 2023 Dec 28;16(1):2295429. doi: 10.1080/19490976.2023.2295429 (PMC10761013; doi:10.1080/19490976.2023.2295429)

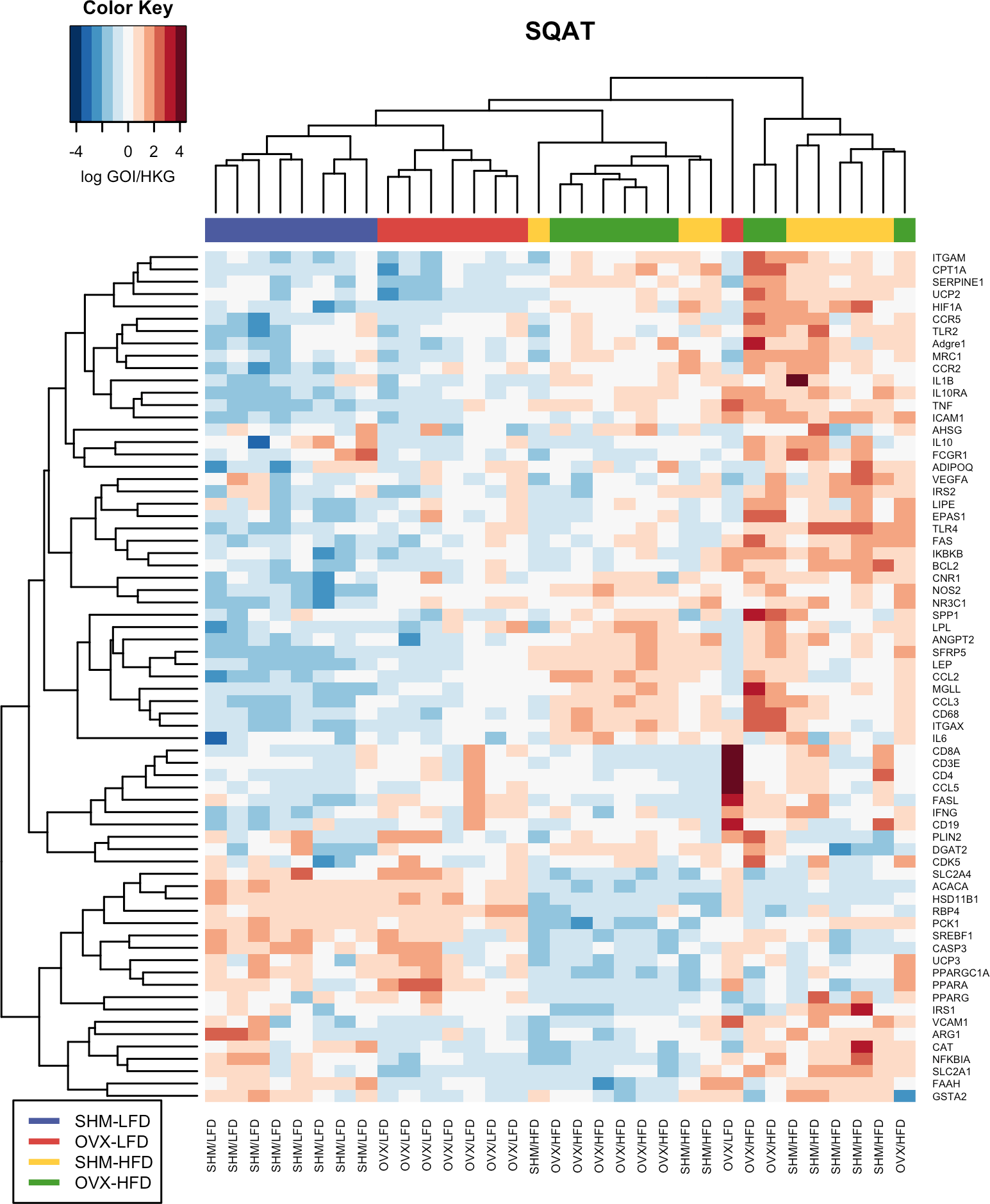

Supplement: SupplementalFig1.docx [file KGMI_A_2295429_SM1878.docx]

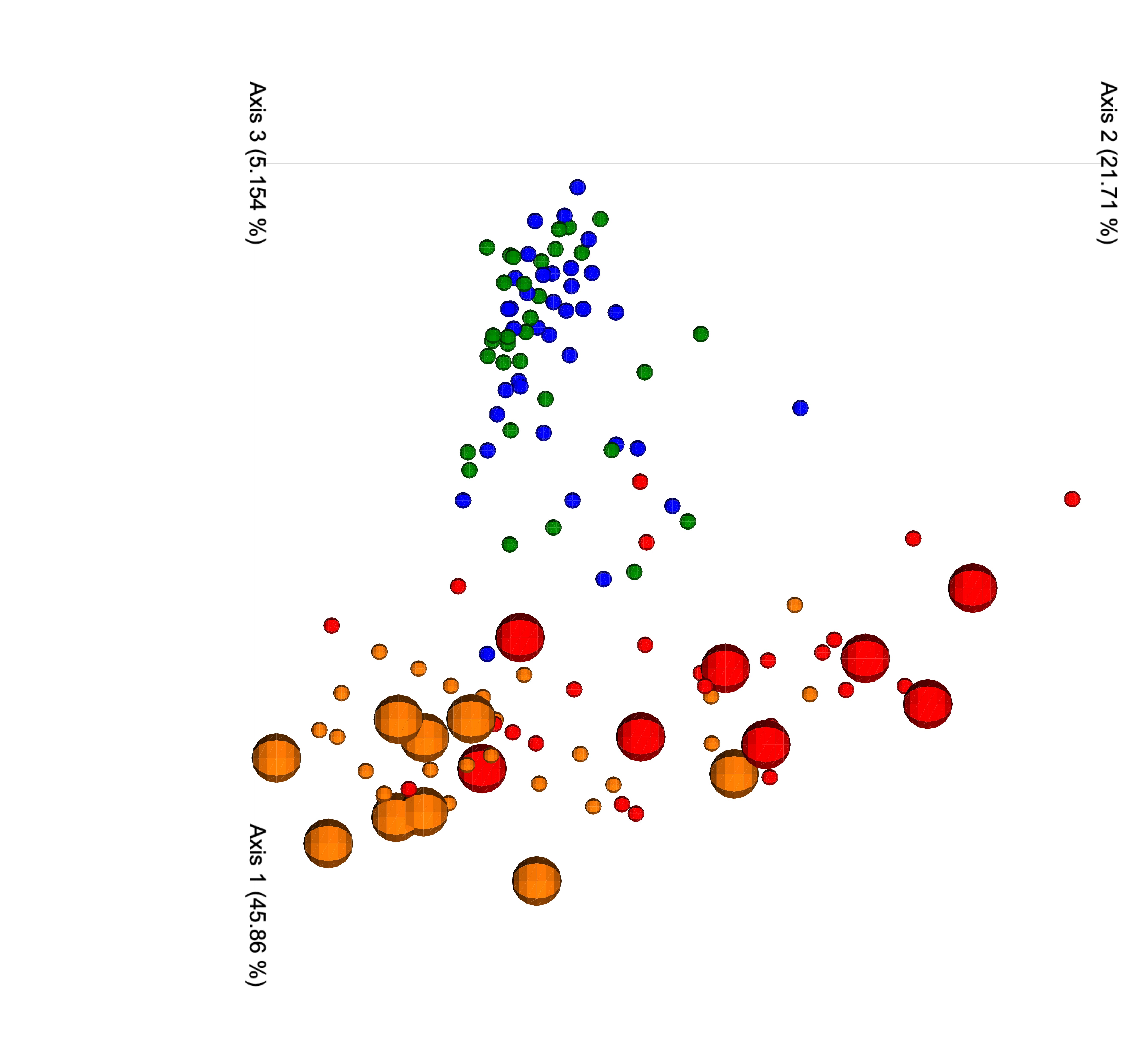
Study timeframe (week)

**HFD**

**LFD**

**0**

**4**

**8**

**12**


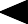


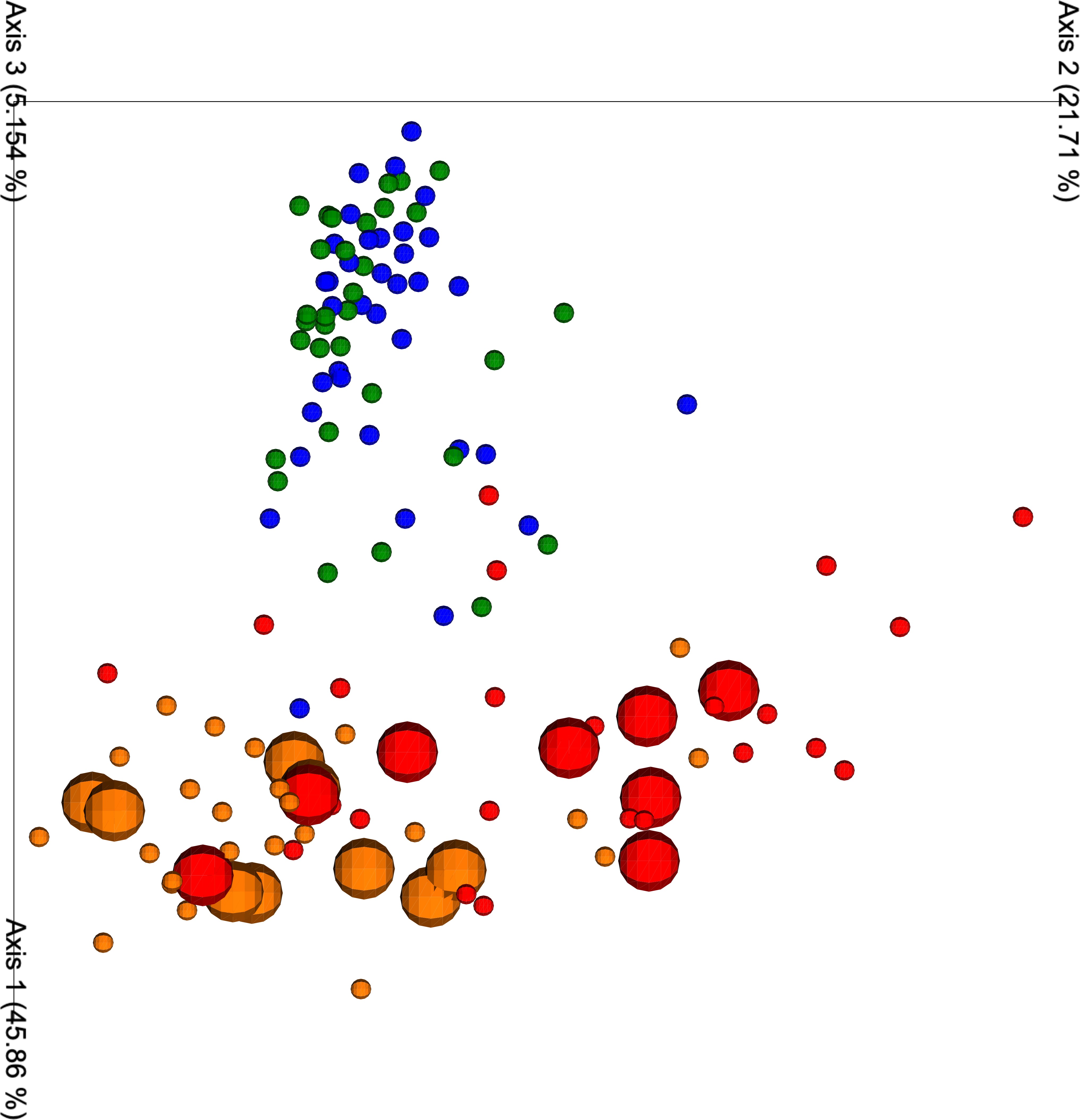

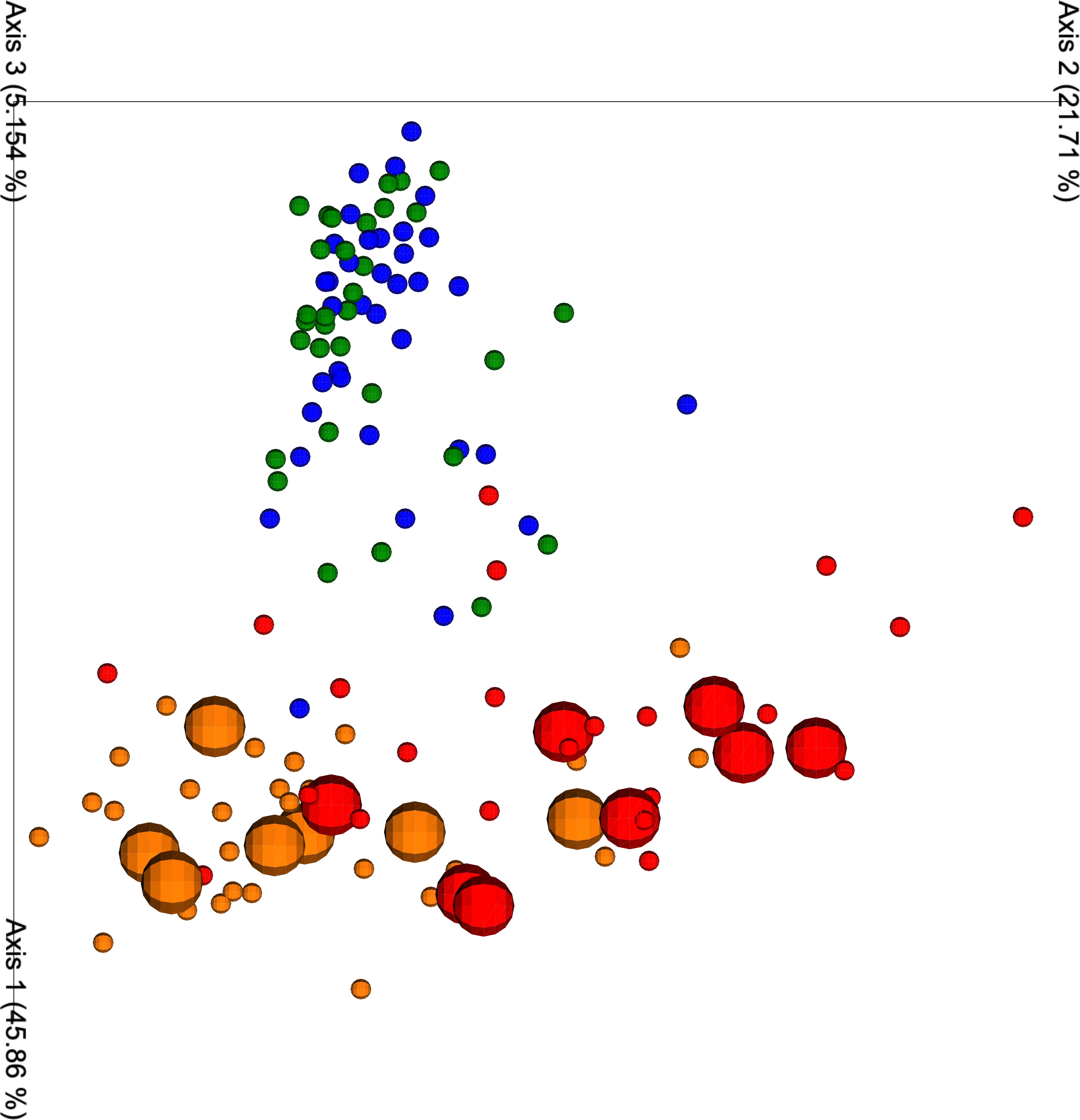

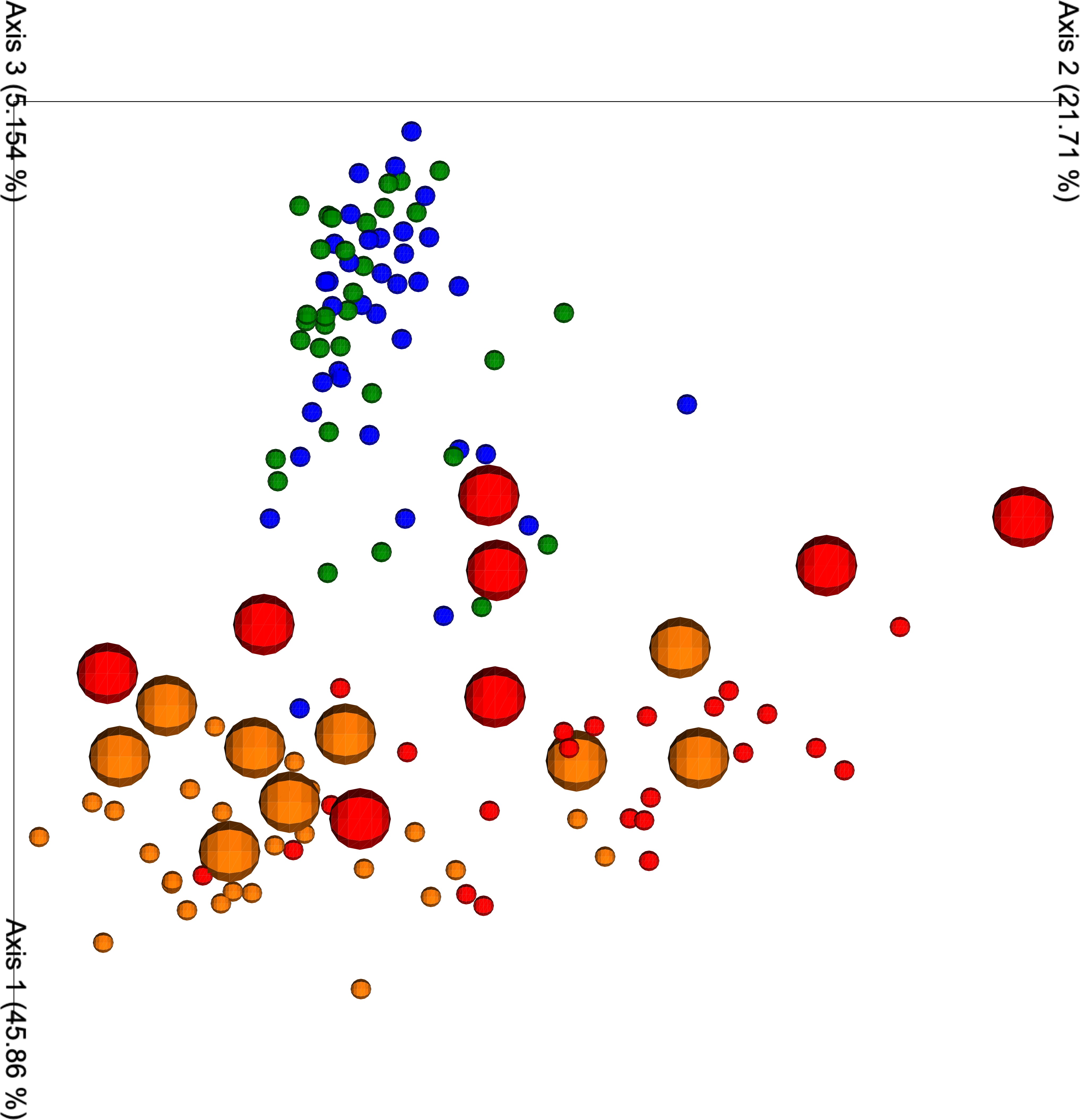


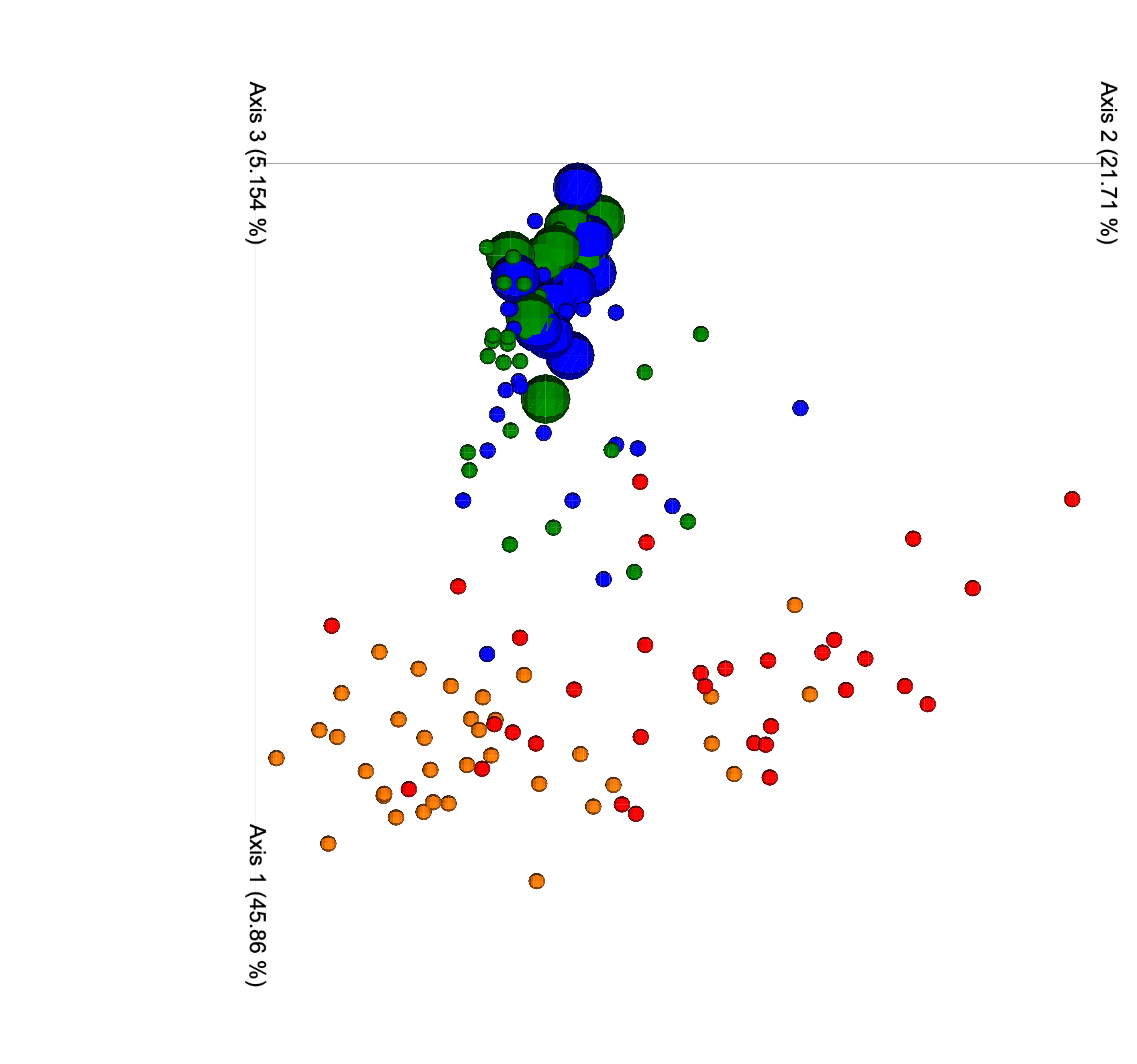

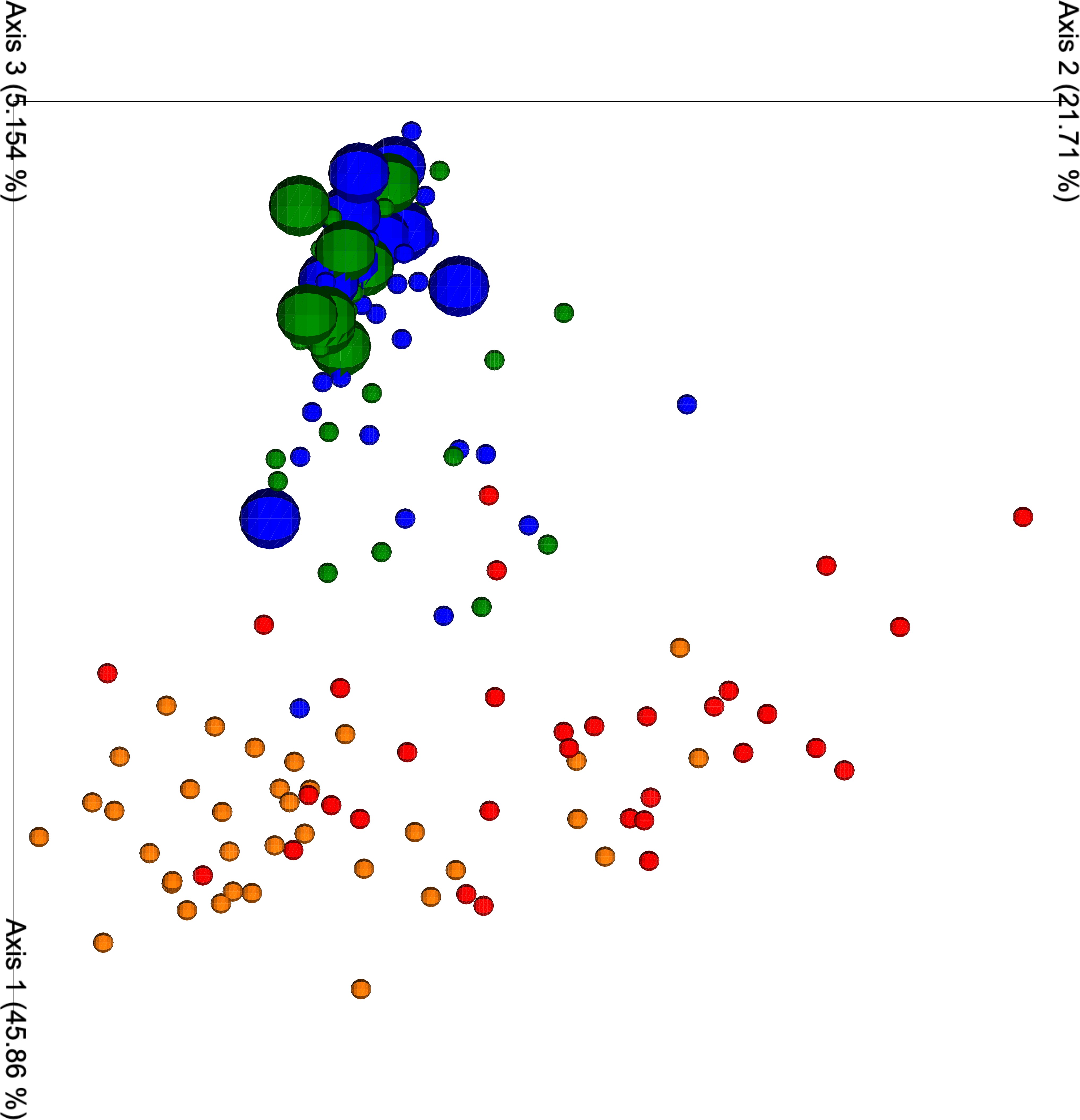

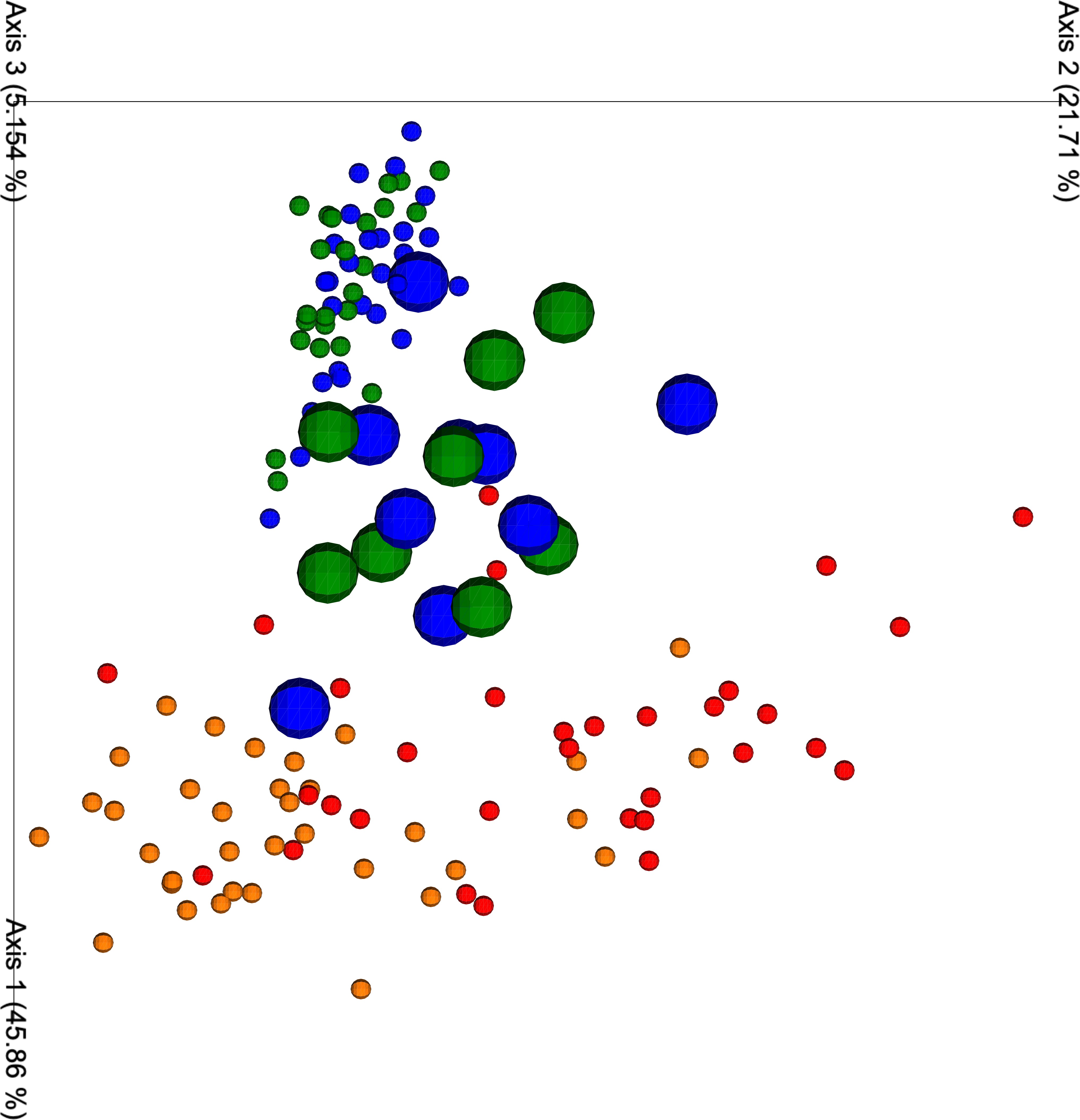

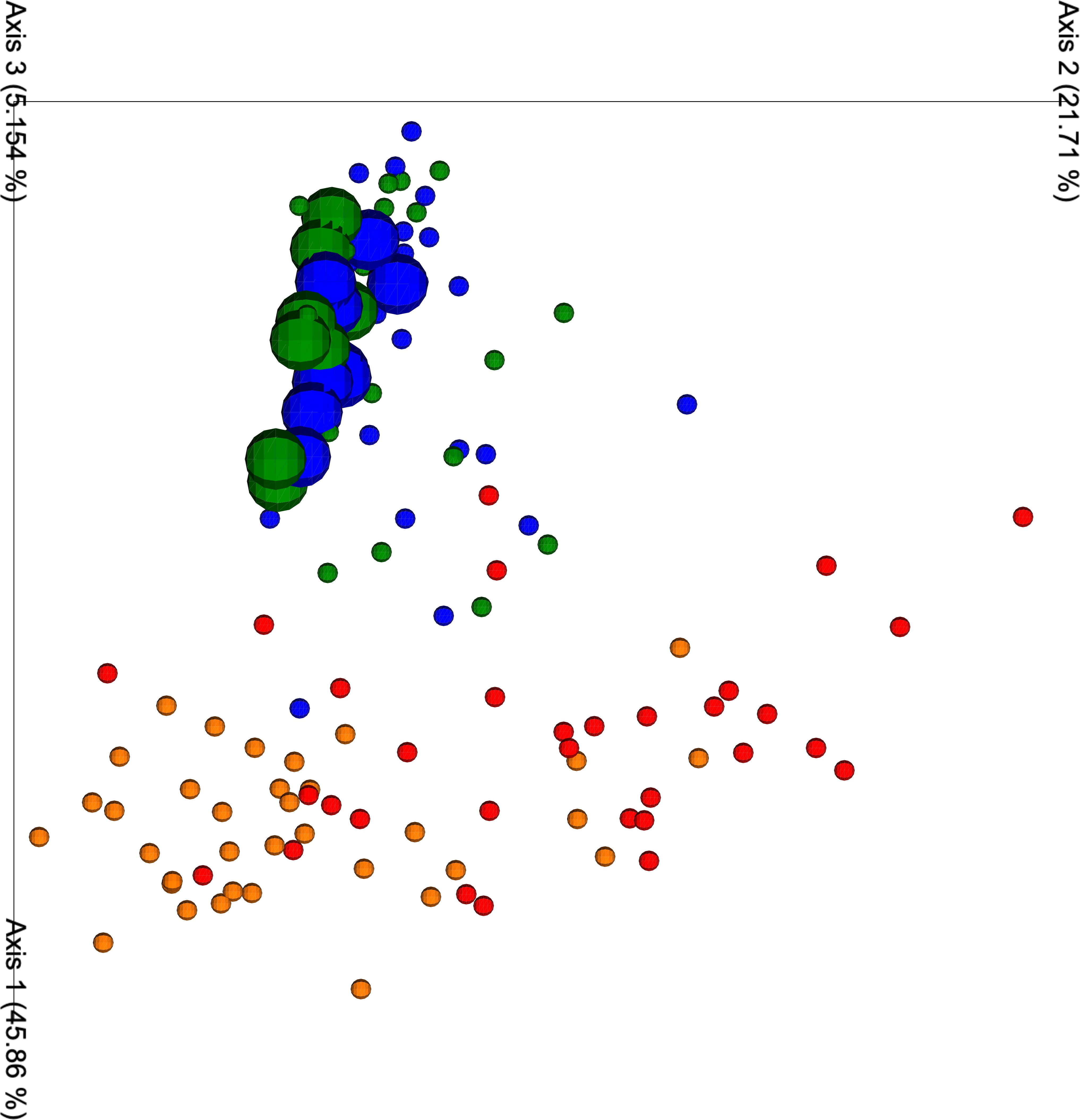

Supplement: SupplementalFig5.docx [file KGMI_A_2295429_SM1876.docx]

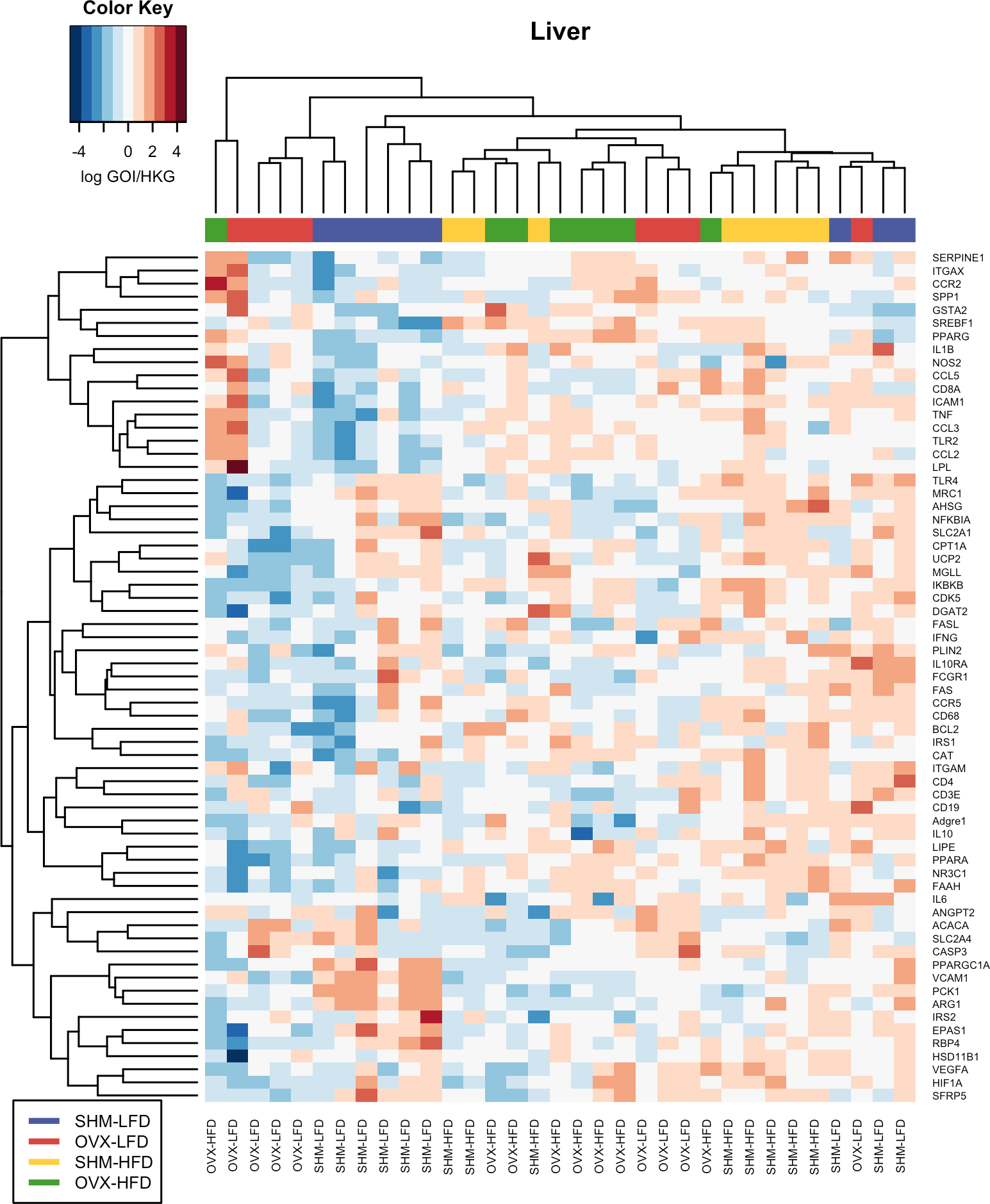

Supplement: SupplementalFig2.docx [file KGMI_A_2295429_SM1875.docx]

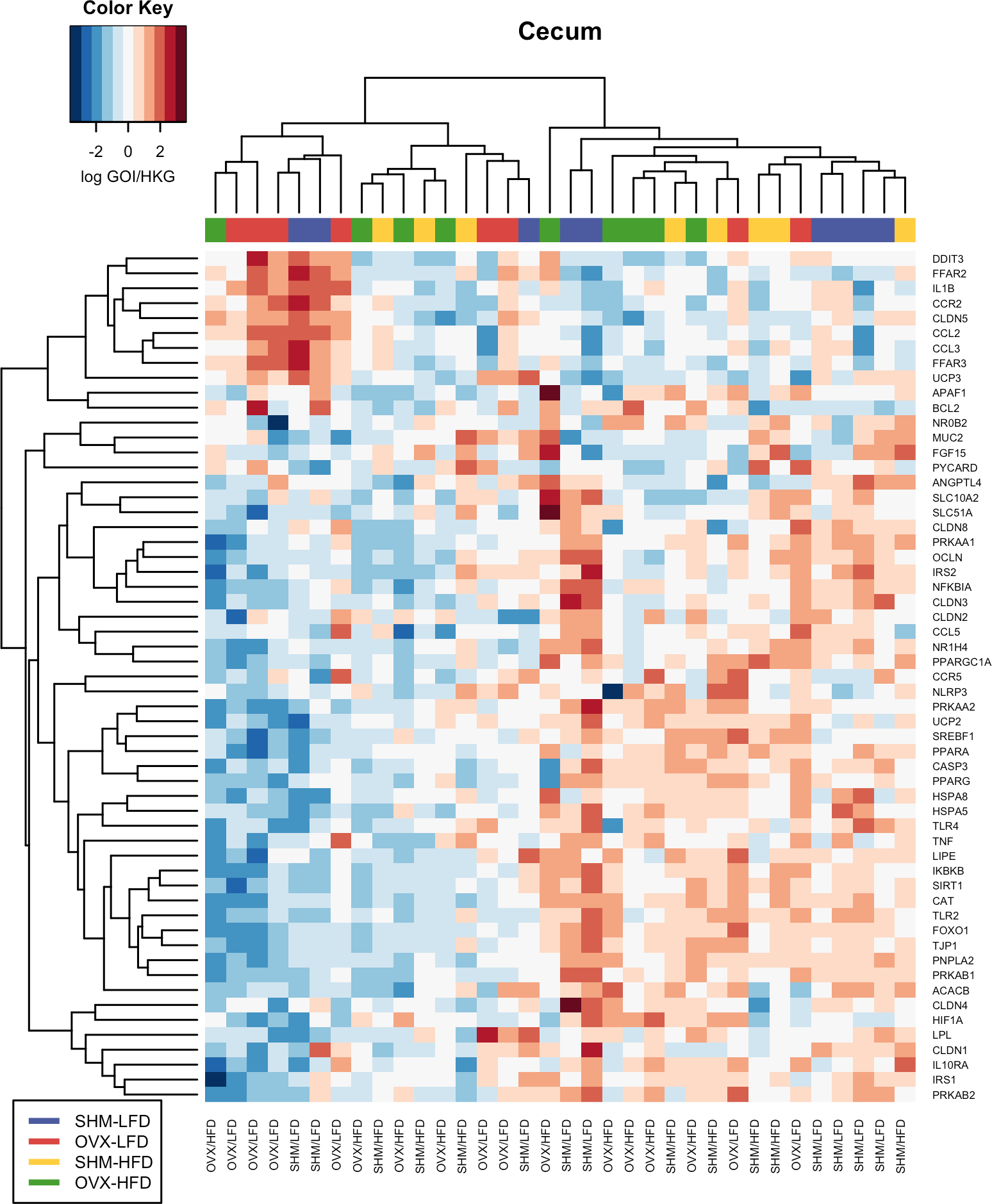

Supplement: SupplementalFig3.docx [file KGMI_A_2295429_SM1873.docx]
