# Supplementary material for: Gut microbiome responds to alteration in female sex hormone status and exacerbates metabolic dysfunction
Source: Gut Microbes. 2023 Dec 28;16(1):2295429. doi: 10.1080/19490976.2023.2295429 (PMC10761013; doi:10.1080/19490976.2023.2295429)
Supplement: SupplementalFig4.docx [file KGMI_A_2295429_SM1874.docx]

**12.0**

**11.9**

**11.8**

**Log10 DNA**

**11.7**

**11.6**

**11.5**

**11.4**

# Universal 16S


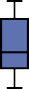

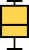

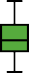


**a**

**b**

**b**

**b**

## SHM OVX SHM OVX LFD HFD

**11.5**

**11.0**

**Log10 DNA**

**10.5**

**10.0**

# Firmicutes


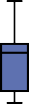

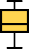

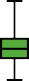


**a**

**a**

**b**

**ab**

## SHM OVX SHM OVX LFD HFD

**10.5**

**10.0**

**Log10 DNA**

**9.5**

**9.0**

# Bacteroidetes


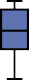

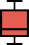


*****

## SHM OVX SHM OVX LFD HFD

**8.0**

**7.5**

**Log10 DNA**

**7.0**

**6.5**

# Rumincoccaceae

**a**

**b**

**b**

**ab**

## SHM OVX SHM OVX LFD HFD

# Lactobacillus

**9**


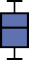

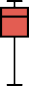

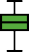


**+**

**+**

**8**

**Log10 DNA**

**7**

**6**

**5**

# Bifidobacterium

**9**


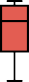

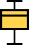

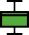


**a**

**b**

**c**

**bc**

**8**

**Log10 DNA**

**7**

**6**

**5**

**9.0**

**8.5**

**Log10 DNA**

**8.0**

**7.5**

**7.0**

# Blautia

**6.5**


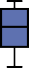

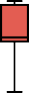

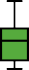


**6.0**

**Log10 DNA**

**5.5**

**5.0**

# Enterococcus

**4**

## SHM OVX SHM OVX LFD HFD

**4**

## SHM OVX SHM OVX LFD HFD

**6.5**

## SHM OVX SHM OVX LFD HFD

**4.5**

## SHM OVX SHM OVX LFD HFD

# Faecalibacterium

**8**


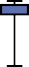

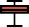

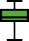


**6**

**Log10 DNA**

**4**

**2**

**0**

## SHM OVX SHM OVX LFD HFD

**4.0**

**3.8**

**3.6**

**Log10 DNA**

**3.4**

**3.2**

**3.0**

**2.8**

# Streptococcus


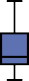

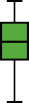


*****

## SHM OVX SHM OVX LFD HFD

**10**

**8**

**Log10 DNA**

**6**

**4**

**2**

**0**

# Turicibacter


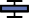

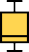

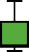


*****

## SHM OVX SHM OVX LFD HFD

**8.5**

**8.0**

**Log10 DNA**

**7.5**

**7.0**

**6.5**

# Fusobacterium


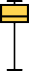


## SHM OVX SHM OVX LFD HFD
